# Plant Glucosinolate Content and Host-Plant Preference and Suitability in the Small White Butterfly (Lepidoptera: Pieridae) and Comparison with Another Specialist Lepidopteran

**DOI:** 10.3390/plants12112148

**Published:** 2023-05-29

**Authors:** Francisco Rubén Badenes-Pérez

**Affiliations:** 1 Max Planck Institute for Chemical Ecology, Department of Entomology, 07745 Jena, Germany; fr.badenes@csic.es; 2 Instituto de Ciencias Agrarias, Consejo Superior de Investigaciones Científicas, 28006 Madrid, Spain

**Keywords:** abaxial leaf side, adaxial leaf side, Brassicales, cabbage white, diamondback moth, glucosinolate diversity, imported cabbageworm, oviposition, *Pieris rapae*, *Plutella xylostella*

## Abstract

Glucosinolates are used in host-plant recognition by insects specialized on Brassicaceae, such as *Pieris rapae* L. (Lepidoptera: Pieridae). This research investigated the association between *P. rapae* oviposition and larval survival and host-plant glucosinolate content using 17 plant species in which glucosinolate content had previously been determined. Two-choice oviposition tests (comparing each plant species to *Arabidopsis thaliana* L.) and larval survival experiments showed that indolic glucosinolate content had a positive effect on oviposition preference and larval survival in *P. rapae*. In the host plants tested, the effects of indolic glucosinolates on oviposition preference and of glucosinolate complexity index and aliphatic glucosinolates without sulfur-containing side chains on total oviposition were smaller on *P. rapae* than on *Plutella xylostella* L. (Lepidoptera: Plutellidae), another lepidopteran specialized on glucosinolate-containing plants. This study suggests that high indolic glucosinolate content could make crop plants more susceptible to both *P. rapae* and *P. xylostella*, but this effect seems to be greater for *P. xylostella*. Additionally, as some differences in oviposition and larval survival between *P. rapae* and *P. xylostella* occurred in some individual plants, it cannot be concluded that bottom-up factors are always similar in these two specialist insects.

## 1. Introduction

Some plant orders are characterized by secondary metabolites that do not occur in other plants [1]. Plants in the order Brassicales typically contain glucosinolates, which are used, among other functions, for plant defense [2,3,4]. The main defense mechanism provided by glucosinolates occurs when they are hydrolized by myrosinases upon plant damage, producing isothiocyanates and other compounds that can be toxic to insects [5,6]. However, larvae of the small white butterfly *Pieris rapae* L. (Lepidoptera: Pieridae), also known as the imported cabbageworm, possess a nitrile-specifier protein that directs glucosinolate hydrolysis to the formation of the less toxic nitriles [7,8,9]. Glucosinolates can also act as feeding stimulants for larvae of *P. rapae* [10,11,12,13]. Compared to plants with lower glucosinolate content, plants with higher glucosinolate content have been shown to have more damage by larvae of *P. rapae* [14]. However, experiments conducted with *Arabidopsis thaliana* (L.) Heynh. and *Brassica oleracea* L. (Brassicaceae) found that some aliphatic glucosinolates can have a negative effect on the growth of *P. rapae* larvae [15,16], and in the case of *B. oleracea*, this effect can be influenced by plant age [16]. Additionally, a study found that allyl isothiocyanate, derived from the aliphatic glucosinolate sinigrin, reduced survival and growth in *P. rapae* larvae [17]. Another study conducted with *B. oleracea* found an association between the content of the indolic glucosinolate neoglucobrassicin and slower development of *P. rapae* larvae [18]. Other studies have shown no clear relationship between glucosinolate content and the presence, preference, and performance of *P. rapae* [19,20,21,22].

Glucosinolates can also act as host recognition cues for *P. rapae* prior to ovipositing on plants [10,23,24,25]. Even pure individual glucosinolates, such as allylglucosinolate, have been shown to stimulate oviposition in *P. rapae* [26]. Different glucosinolates can also stimulate oviposition differently, and *P. rapae* prefers indol-3-ylmethylglucosinolate and 2-phenylethylglucosinolate over allylglucosinolate [23,27,28]. On the other hand, indole-3-acetonitrile, which is derived from indol-3-ylmethylglucosinolate and can be present in the regurgitant of *P. rapae* larvae, can be an oviposition deterrent [29]. When comparing plants of the same species with different glucosinolate content, *P. rapae* also preferred to oviposit on lines with higher concentrations of total glucosinolates and lower concentrations of certain aliphatic glucosinolates [10,30,31].

Studies addressing the association between host-plant glucosinolate content and preference and suitability for *P. rapae* have been conducted comparing plants of the same or closely-related species, such as *A. thaliana* and *B. oleracea*. No previous studies have used a wide variety of plant species to investigate the effect of plant glucosinolate content on the plant preference and suitability for *P. rapae*. Here, 17 plant species containing a wide range of glucosinolates were used with the purpose of testing whether oviposition preference and larval survival were affected by glucosinolate content in *P. rapae*. The other objective of this research was to compare the oviposition preference and larval survival of *P. rapae* to previously determined oviposition preference and larval survival values of the diamondback moth, *Plutella xylostella* L. (Lepidoptera: Plutellidae), on the same plant species. The purpose of comparing these two lepidopteran species that use glucosinolate-containing plants as their host plants was to test if these two specialists showed a similar response to plant glucosinolate content. *Pieris rapae* can be an economic pest in cruciferous crops, although usually it is not as significant as a pest as *P. xylostella* [32,33,34,35,36]. Knowledge on the association between host-plant glucosinolate content and oviposition and suitability can be used in host-plant resistance and trap cropping strategies to reduce the damage caused by these lepidopterans.

## 2. Results

### 2.1. Two-Choice Oviposition Preference Tests

There were some significant differences in the oviposition preference index of *P. rapae* in the plants tested, both when considering the plants individually (the plant species compared to *A. thaliana*) (Table 1) as well as when comparing the oviposition preference index values among the different plant species (Table S1). Except for *B. orientalis*, values of oviposition preference index for *P. rapae* were below 1, indicating that *A. thaliana* would tend to be preferred. This trend for significant preference for *A. thaliana* was statistically confirmed in the comparisons with 11 of the plant species tested (Table 1). For the plant species tested, the only significant difference between the oviposition preference index values of *P. rapae* and *P. xylostella* occurred in the comparison with *L. douglasii*, on which *P. rapae* females did not oviposit at all, while *P. xylostella* showed a relatively high oviposition preference index value of 3.84 for this plant species (Table 1 and Table S2).

For the set of plants tested, the oviposition preference indexes of *P. rapae* and *P. xylostella* were not significantly correlated (Table S3). However, oviposition preference index was positively correlated with total oviposition in *P. rapae* and with total oviposition and larval survival in *P. xylostella*. The glucosinolate content and glucosinolate diversity indexes of the plants tested are shown in Tables S4 and S5. In both *P. rapae* and *P. xylostella*, indolic glucosinolate content had a significant effect on oviposition preference index (Table 2 and Table S6; Figure S1A). As shown in Table 2 by the negative value of the B regression coefficient for *P. rapae*, the effect of indolic glucosinolate content on oviposition preference index was greater for *P. xylostella* than for *P. rapae*. 

### 2.2. No-Choice Oviposition Tests

*Pieris rapae* showed significant differences in total oviposition (*p* ≤ 0.001) across the plants tested; total oviposition was highest for *C. spinosa*, *B. oleracea*, and *C. pratensis* and lowest for *C. papaya*, *L. douglasii*, *M. oleifera*, and *P. sativum* on which no oviposition occurred (Table 3 and Table S7). When comparing the total oviposition of *P. rapae* and *P. xylostella*, there were significant differences between these two species for *A. argenteum*, *A. caucasica*, *B. oleracea*, *C. bursa-pastoris*, *I. amara*, *L. douglasii*, *M. oleifera*, *P. sativum*, and *R. odorata* (Table 3). On *A. argenteum*, *A. caucasica*, *C. bursa-pastoris*, *I. amara*, *L. douglasii*, *M. oleifera*, and *P. sativum*, total oviposition was higher for *P. xylostella* than for *P. rapae*, while in the case of *B. oleracea* and *R. odorata*, total oviposition was higher for *P. rapae* than for *P. xylostella*.

There was a significant positive correlation between total oviposition and larval survival in both *P. rapae* and *P. xylostella* (Table S3). For the set of plants tested, in both *P. rapae* and *P. xylostella*, the content of aliphatic glucosinolates without sulfur-containing side chains, glucosinolate complexity index, and Shannon’s diversity index for the four glucosinolate classes had a significant effect on total oviposition (Table 2 and Table S6; Figure S1B). As shown in Table 2 by the negative value of the B regression coefficient for *P. rapae*, the overall effects on total oviposition of aliphatic glucosinolates without sulfur-containing side chains, glucosinolate complexity index, and Shannon’s diversity index for the four glucosinolate classes were greater for *P. xylostella* than for *P. rapae*.

### 2.3. Abaxial vs. Adaxial Oviposition Preference

*Pieris rapae* preferred to oviposit on the adaxial leaf side in the case of *A. thaliana* and *I. amara* (Table 4). For the other plant species tested, the differences between abaxial and adaxial oviposition were not significant. The only significant difference in abaxial versus adaxial oviposition between *P. rapae* and *P. xylostella* occurred in *A. thaliana*, on which oviposition was mostly adaxial for *P. rapae*, while *P. xylostella* showed no significant preference for either leaf side on this plant species (Table S8).

### 2.4. Larval Survival Experiments

*Pieris rapae* showed significant differences in larval survival (*p* ≤ 0.001) across the plants tested. Survival was highest on *B. oleracea*, *C. pratensis*, *R. odorata*, *A. thaliana*, and *B. vulgaris* and lowest on the rest of the plants tested, in which no larvae survived (Table 5 and Table S9). When comparing the larval survival of *P. rapae* and *P. xylostella*, there were significant differences between these two species for *L. douglasii*, on which larvae of *P. rapae* did not survive, while the survival of *P. xylostella* was 66.7% (Table 5).

On the group of plants tested there was a highly significant positive correlation between larval survival and total oviposition in *P. rapae*, but this correlation was not significant when comparing larval survival and oviposition preference index (Table S3). Larval survival in *P. rapae* was also positively correlated with indolic glucosinolate content and glucosinolate complexity index. In *P. xylostella*, larval survival was only positively correlated with total oviposition. For the set of plants tested, in both *P. rapae* and *P. xylostella*, the content of indolic glucosinolates without sulfur-containing side chains and glucosinolate complexity index had a significant effect on total oviposition (Table 2 and Table S6; Figure S1C). These effects of glucosinolate content on larval survival were not significantly different between *P. xylostella* and *P. rapae* (Table 2).

## 3. Discussion

This research shows that for *P. rapae* and *P. xylostella*, oviposition preference index and larval survival values were positively affected by indolic glucosinolate content. Glucosinolate complexity index also affected larval survival in these two insects specialized on glucosinolate-containing plants. Aliphatic glucosinolates without sulfur-containing side chains, glucosinolate complexity index, and one of the Shannon’s diversity indexes considered had a significant effect on total oviposition. The overall effects of glucosinolate content on the oviposition preference index and total oviposition were greater on *P. xylostella* than on *P. rapae*. Although some differences on individual plants occurred, oviposition preference index values were positively correlated in *P. rapae* and *P. xylostella* but total oviposition and larval survival were not. This indicates that similarities occur between these two specialists, but there are also differences in oviposition and host-plant suitability.

The plants included in this study comprised a wide range of glucosinolates (32 in total), more than one fourth of the number of glucosinolates characterized from plants so far, which has been estimated to be somewhere between 88 and 137 [39]. However, glucosinolates are not the only factors affecting oviposition and herbivory in *P. rapae*. Phenolic acids and other plant metabolites can affect *P. rapae* oviposition [40,41]. Plant color, nitrogen and phosphorous content, environmental conditions, presence of conspecific larvae, and the physiological status of the insects can also influence *P. rapae* oviposition [42,43,44,45,46,47,48]. Spatial factors can also be important in *P. rapae* oviposition, as they tend to lay more eggs on isolated host plants [49,50]. Females of *P. rapae* use both olfactory and visual cues in host selection [51,52]. Pre-alighting behavior of *P. rapae* females in the field seems to involve passing over many suitable host plants and the spreading of eggs [42,50]. When comparing plants of the same species, ovipositing *P. rapae* seems to prefer larger plants [53,54]. Populations from different locations can differ in their host search and host selection behavior [55]. Cucurbitacins in species such as *I. amara* and cardenolides in some *Erysimum* spp. can be feeding deterrents for *P. rapae* larvae [56,57]. The presence of feeding deterrents has often been linked to host-plant unsuitability for *P. rapae* [56,57,58]. Unlike *P. xylostella*, whose larvae cannot survive on G-type *B. vulgaris* [59,60], *P. rapae* has a relatively high survival rate on this plant species.

Differences in abaxial versus adaxial leaf side oviposition occurred in some plant species for *P. rapae*. In two of the plant species tested, differences in abaxial versus adaxial oviposition were different for *P. rapae* and *P. xylostella*. Abaxial versus adaxial oviposition preference may affect the management of these insects. For example, some insecticide sprayers deposit more insecticide on the adaxial than on the abaxial leaf side [61], and larval parasitism is higher for *P. rapae* larvae located on the adaxial side of *B. oleracea* leaves [62]. In *P. xylostella*, it has been shown that egg susceptibility to rainfall is greater on the abaxial leaf side [63].

For the plant species tested here, the correlation between oviposition preference index and larval survival was not significant for either *P. rapae* or *P. xylostella*, although in the case of *P. rapae*, this correlation was almost significant. Another study also showed that some plant genotypes of *A. thaliana* had opposite effects on oviposition preference and larval performance in *P. rapae* [30]. However, in different sets of plants tested, the correlation between preference and performance was significant for these two herbivores [37,64,65]. The correlation between preference and performance, also referred to as ‘the mother knows best principle’, is considered to be stronger in oligophagous insects [66], such as *P. rapae* and *P. xylostella*.

The larval survival results shown here indicate that *P. xylostella* might have a broader host range than *P. rapae*. This could be due to *P. xylostella* being less selective when accepting host plants for oviposition and having more efficient detoxification means. In terms of glucosinolate detoxification, the mechanisms used by *P. xylostella* and *P. rapae* are different [7,8,9,67]. Some studies have shown that *P. rapae* larvae can be negatively affected by glucosinolates and their hydrolysis products [15,16,17], indicating that glucosinolate detoxification in *P. rapae* could be less effective than in *P. xylostella*. 

*Pieris brassicae* L., a species closely related to *P. rapae*, is also found in association with glucosinolate-containing plants, and indolic glucosinolates also act as oviposition stimulants for this species [68,69,70].

This study did not compare the effect of individual glucosinolates on *P. rapae* oviposition and LS. However, in studies involving different lines of *B. oleracea* with different concentrations of individual glucosinolates, the content of certain individual glucosinolates has been associated with feeding suitability and abundance of *P. rapae* larvae [71]. As glucosinolates can be induced as a result of herbivory, including feeding by *P. rapae* larvae [72,73], glucosinolate content could have changed during the larval survival tests compared to the glucosinolate content of intact plants.

Even though glucosinolates can provide resistance against generalist herbivores and lengthen the development time of generalist larvae [74,75,76], in areas where the prevalent insect pests are the specialists *P. rapae* and *P. xylostella*, the use of crop varieties with low IN content could reduce insect damage. On the other hand, the preferential oviposition preference of *P. rapae* for plants with higher indolic glucosinolate content could be used in the selection of trap crops, which, unlike in *P. xylostella*, have so far not been tested successfully in the management of *P. rapae* [35,77,78,79].

## 4. Materials and Methods

### 4.1. Plant Growth, Glucosinolate Content, and P. rapae Culture

Among the 17 plant species tested, 10 belonged to 7 different subfamilies within the family Brassicaceae (order Brassicales), 6 belonged to 6 other families in the order Brassicales, and 1 (*Pisum sativum* L. cultivar Oregon Sugar Pod) belonged to the family Fabaceae (order Fabales) and was used as a control because it does not contain glucosinolates and is not a host plant for *P. rapae* (Table 6). For the different plant species used, the origin of the seeds can be found in Table S10. In natural conditions, these plant species overlap with *P. rapae* butterflies during the time of the year that females are actively searching for host plants to oviposit. Among the plants tested are some known to be highly attractive and suitable for *P. rapae*, such as *B. vulgaris*, *B. oleracea*, and *C. pratensis* [22,64,80]; some known to be poor hosts for *P. rapae*, such as *B. orientalis*, *C. bursa-pastoris*, and *T. majus* [23,64,81,82,83]; and some that have not previously been tested as host plants for this insect, such as *C. papaya*, *L. douglasii*, and *M. oleifera*. In addition to total glucosinolate content, four different classes of glucosinolates were distinguished: aliphatic with sulfur-containing side chains, other aliphatic, benzenic, and indolic. Glucosinolate diversity was analyzed taking into account the glucosinolate richness, Shannon’s diversity index for the four glucosinolate classes, Shannon’s diversity index for the relative concentrations of all individual glucosinolates, and glucosinolate complexity index for each plant [37]. *Arabidopsis thaliana* plants were grown in a climate chamber (10:14 h light/dark, 21 ± 2 °C and 55 ± 5 RH), and the other plant species were grown in a greenhouse (16:8 h light/dark, 25 ± 3 °C). Plants were grown in 7 × 7 × 8-cm pots using peat moss substrate with clay. All plants used in the experiments were 5 to 6 weeks old at the beginning of the experiments. The *P. rapae* insects used were collected in Jena, Germany, and were successively reared on cabbage plants. Insects were reared in environmental growth chambers (16:8 h light:dark, 21 ± 2 °C and 55 ± 5 RH).

### 4.2. Two-Choice Oviposition Preference Tests

Two-choice oviposition experiments were conducted in comparison with *A. thaliana* (i.e., one plant of any of the tested types versus one plant of *A. thaliana*) to measure oviposition preference, similarly to previous studies conducted with *P. xylostella* [42]. *Arabidopsis thaliana* was chosen as a reference plant in the two-choice tests because it is the most widely available and used model plant and also because it has also been extensively studied in glucosinolate research. The experimental arenas used consisted of 32.5 × 32.5 × 32.5 cm polyester cages with 96 × 26 mesh (MegaView Science Education Services Co., Ltd., Taichung, Taiwan). Multiple cages were used, each of which was considered a replicate. Two pairs of *P. rapae* butterflies (two females and two males, <3 days old) were released in each cage. To provide a food source for the butterflies, a small plastic cup with a 10% sugar solution on cotton was placed in the middle of each cage. The experiment was replicated at least three times for each plant comparison. Two days after releasing the butterflies, the number of eggs on the plants was counted in the laboratory. An oviposition preference index was calculated as the number of eggs laid on each individual plant divided by the number of eggs laid on the *A. thaliana* plant that it was compared with in the same cage [42]. An oviposition preference index = 1 indicated no difference in oviposition preference between *A. thaliana* and the alternative plant species it was compared with; an oviposition preference index <1 indicated that *A. thaliana* would tend to be preferred; and an oviposition preference index >1 indicated that *P. rapae* would tend to prefer the alternative plant species over *A. thaliana*.

### 4.3. No-Choice Oviposition Tests

Oviposition experiments were conducted as described above for the two-choice oviposition preference experiments but with only 1 single plant of the 17 species tested. Total oviposition on each plant was replicated at least three times.

### 4.4. Abaxial vs. Adaxial Oviposition Preference

The numbers of eggs on the abaxial and adaxial leaf sides of each plant were also recorded in the two-choice and no-choice oviposition preference tests described above in order to determine if *P. rapae* had a particular oviposition preference for either abaxial or adaxial leaf surfaces in the plant species tested.

### 4.5. Larval Survival Experiments

Five first-instar *P. rapae* larvae (<2 d after hatching) were randomly placed on five fully expanded leaves within each plant. The same procedure was repeated on three plants (n = 3) for each plant type. When necessary, in case of extensive defoliation of a plant, larvae were transferred to a new plant of the same age. To prevent larval movement between plants, plants were kept individually in either 32.5 × 32.5 × 32.5 cm cages with 96 × 26 mesh (MegaView Science Education Services Co., Ltd., Taichung, Taiwan) or in larger 61 × 61 × 61 cm cages with 32 × 32 mesh (BioQuip Products, Rancho Dominguez, CA, USA). Larval survival was recorded as the percentage of individuals that reached pupation per plant.

### 4.6. Statistical Analysis

Data comparing insect oviposition preference between the different plant types and *A. thaliana* and between abaxial and adaxial leaf surfaces were analyzed using a one-tailed, two-sample test of proportions using STATA^®^ version 15.1 with significance at *p* ≤ 0.05. Data comparing values of oviposition preference index, abaxial oviposition, and larval survival between *P. rapae* and *P. xylostella* were analyzed using a one-tailed, two-sample test of proportions using STATA^®^. Data comparing total oviposition values on the different plant species between *P. rapae* and *P. xylostella* were analyzed either using ANOVA if the data were parametric or using the Moses test of extreme reactions if the data were non-parametric using SPSS^®^ version 28.0.1.0. Correlations between oviposition and larval survival in *P. rapae* and *P. xylostella* were performed using two-tailed Spearman’s correlations with SPSS^®^. Categorical principal component analysis (CATPCA) was performed with SPSS^®^ to explore the relationships between glucosinolate content and oviposition and larval survival in the two insects tested. After the exploratory use of CATPCA, to confirm the effect of glucosinolates on oviposition and larval survival on the two insect species, a generalized linear model with a Poisson probability distribution with log link function was used by means of the GENLIN procedure of SPSS^®^. Only the variables with values of correlation to oviposition and larval survival above 0.35 in the CATPCA were considered in the GENLIN model. The significance of the variables in the model was assessed using Wald Chi-square tests. Variables that did not have a significant effect were removed from the model. Data comparing *P. rapae* oviposition preference index and larval survival in the different plants tested were analyzed using the Kruskal–Wallis test with SPSS^®^. Prior to GENLIN analysis, the values of oviposition preference index were multiplied by 100 and then rounded to the nearest integer, while the values of total oviposition and larval survival were multiplied by 10 and then rounded to the nearest integer. The rest of the statistical analyses were performed with untransformed data.

## 5. Conclusions

This research shows that for *P. rapae* and *P. xylostella*, oviposition preference index and larval survival values were positively affected by indolic glucosinolate content. Glucosinolate complexity index also affected larval survival and total oviposition in these two insects specialized on glucosinolate-containing plants. Aliphatic glucosinolates without sulfur-containing side chains and one of the Shannon’s diversity indexes considered had a significant effect on total oviposition. The overall effects of glucosinolate content on the oviposition preference index and total oviposition were greater on *P. xylostella* than on *P. rapae*. Individual differences in oviposition and larval survival also occurred between *P. rapae* and *P. xylostella* in some host plants. These indicate that the significance of bottom-up factors is not necessarily similar for these two specialist insects.

## Figures and Tables

**Table 1:** Two-choice oviposition preference index (OPI) in *P. rapae* and *P. xylostella* larvae reared on cabbage. Data were analyzed using a one-tailed, two-sample test of proportions comparing the relative percentages of all eggs laid on the plant being tested and on *A. thaliana* (*p* ≤ 0.05) (n = 3). OPI and percentage of eggs on the plant tested compared to *A. thaliana* given as means found across replicates (mean ± SE). Significant differences are shown in bold type. *Plutella xylostella* data taken from Badenes-Pérez et al. [37].

	OPI, % Eggs on Plant Species Tested Compared to *A. thaliana*, Test Statistic, and *p*-Value	
	*P. rapae*	*P. xylostella*
*A. argenteum*	0.01 ± 0.00, 0.56 ± 0.40, *z* = 2.42, *p* = 0.008 *	0.08 ± 0.02, 7.25 ± 1.38, *z* = 2.11, *p* = 0.018 *
*A. caucasica*	0.04 ± 0.03, 3.70 ± 2.62, *z* = 2.27, *p* = 0.012 *	0.43 ± 0.05, 29.79 ± 2.52, *z* = 0.99, *p* = 0.161
*B. vulgaris*	0.61 ± 0.13, 36.04 ± 5.75, *z* = 0.68, *p* = 0.247	2.70 ± 0.99, 69.51 ± 6.49, *z* = 0.96, *p* = 0.169
*B. oleracea*	0.19 ± 0.02, 15.89 ± 1.35, *z* = 1.67, *p* = 0.047 *	0.24 ± 0.06, 18.74 ± 3.68, *z* = 1.53, *p* = 0.063
*B. orientalis*	1.04 ± 0.04, 50.90 ± 0.87, *z* = 0.04, *p* = 0.483	0.18 ± 0.10, 13.99 ± 7.06, *z* = 1.76, *p* = 0.039 *
*C. bursa-pastoris*	0.02 ± 0.01, 1.48 ± 1.05, *z* = 2.38, *p* = 0.009 *	0.03 ± 0.03, 3.19 ± 2.90, *z* = 2.29, *p* = 0.011 *
*C. pratensis*	0.26 ± 0.03, 20.41 ± 1.65, *z* = 1.45, *p* = 0.074	0.71 ± 0.16, 40.48 ± 6.21, *z* = 0.47, *p* = 0.320
*C. papaya*	0.02 ± 0.01, 1.85 ± 1.31, *z* = 2.36, *p* = 0.009 *	0.05 ± 0.05, 4.08 ± 4.08, *z* = 2.25, *p* = 0.012 *
*C. spinosa*	0.39 ± 0.27, 18.32 ± 12.39, *z* = 1.55, *p* = 0.060	0.09 ± 0.05, 7.70 ± 4.60, *z* = 2.07, *p* = 0.019 *
*E. cheiri*	0.03 ± 0.01, 3.24 ± 1.22, *z* = 2.29, *p* = 0.011 *	0.22 ± 0.18, 14.90 ± 11.00, *z* = 1.72, *p* = 0.043 *
*I. amara*	0.07 ± 0.03, 6.48 ± 2.36, *z* = 2.13, *p* = 0.017 *	0.72 ± 0.46, 34.02 ± 15.28, *z* = 0.78, *p* = 0.217
*L. douglasii*	0 ± 0, 0 ± 0, *z* = 2.45, *p* = 0.007 *	3.84 ± 0.86, 77.64 ± 4.73, *z* = 1.35, *p* = 0.088
*M. oleífera*	0 ± 0, 0 ± 0, *z* = 2.45, *p* = 0.007 *	0 ± 0, 0 ± 0, *z* = 2.45, *p* = 0.007 *
*P. sativum*	0.01 ± 0.01, 1.03 ± 0.73, *z* = 2.40, *p* = 0.008 *	0 ± 0, 0 ± 0, *z* = 2.45, *p* = 0.007 *
*R. odorata*	0.46 ± 0.03, 31.42 ± 1.45, *z* = 0.91, *p* = 0.181	0.36 ± 0.30, 20.37 ± 15.58, *z* = 1.45, *p* = 0.073
*T. majus*	0 ± 0, 0 ± 0, *z* = 2.45, *p* = 0.007 *	0.04 ± 0.04, 6.06 ± 6.06, *z* = 2.15, *p* = 0.016 *

* *A. thaliana* preferred.

**Table 2:** Effect of plant glucosinolate content, glucosinolate diversity, and type of specialist lepidopteran (either *P. rapae* or *P. xylostella*) on two-choice oviposition preference index (OPI), no-choice total oviposition (TO), and larval survival (LS) in *P. rapae* and *P. xylostella*. The variables selected and included in the model after CATPCA analysis were indolic glucosinolates (IN), aliphatic glucosinolates without sulfur-containing side chains (AO), Shannon’s diversity index for the four glucosinolate classes (H_A_), and glucosinolate complexity index (GCI). The generalized linear model used was based on a Poisson probability distribution with log link function (*p* ≤ 0.05) (n = 16 for OPI and TO and n = 17 for LS). The regression coefficient B was set to zero for *P. xylostella*. Significant *p*-values are shown in bold type.

	B	Standard Error	Wald Chi Square	*p*
OPI				
Intercept	3.79	0.04	9242.44	**≤0.001**
*P. rapae*	−0.82	0.06	199.04	**≤0.001**
*P. xylostella*	0			
IN	0.18	0.01	321.29	**≤0.001**
TO				
Intercept	5.00	0.03	37,794.14	**≤0.001**
*P. rapae*	−0.648	0.02	805.56	**≤0.001**
*P. xylostella*	0			
H_A_	−0.23	0.07	12.23	**≤0.001**
GCI	0.67	0.03	445.03	**≤0.001**
AO	0.03	0.00	1606.37	**≤0.001**
LS				
Intercept	0.21	0.26	0.65	0.420
*P. rapae*	−0.28	0.23	1.52	0.217
*P. xylostella*	0			
IN	0.12	0.05	6.38	**0.012**
GCI	0.47	0.17	7.84	**0.005**

The *p*-values of the generalized linear model used for OPI, TO, and LS were highly significant (*p* ≤ 0.001) based on Omnibus tests.

**Table 3:** Total oviposition (TO) in non-choice tests (mean ± SE) for each of the tested plants for *P. rapae* and *P. xylostella* reared on cabbage. Differences in TO between *P. rapae* and *P. xylostella* for each plant species were analyzed using either ANOVA (parametric data) or Moses test of extreme reactions (non-parametric data) (*p* ≤ 0.05) (n = 3). Significant differences are shown in bold type. *Plutella xylostella* data taken from Badenes-Pérez et al. [37].

	Number of Eggs (Mean ± SE)		
	*P. rapae*	*P. xylostella*	Test Statistic and *p*-Value
***A. argenteum***	**0.33 ± 0.33**	**91.00 ± 23.69**	**TS = 3.00, *p* ≤ 0.001**
***A. caucasica***	**5.33 ±2.03**	**63.00 ± 11.27**	***F* = 25.36, *p* = 0.007**
*B. vulgaris*	25.33 ± 6.69	44.67 ± 10.68	*F* = 2.35, *p* = 0.200
***B. oleracea***	**69.67 ± 2.91**	**34.33 ± 6.39**	***F* = 25.36, *p* = 0.007**
*B. orientalis*	16.33 ± 6.94	22.67 ± 7.17	*F* = 0.40, *p* = 0.560
***C. bursa-pastoris***	**0.33 ± 0.33**	**15.33 ± 2.91**	**TS = 3.00, *p* ≤ 0.001**
*C. pratensis*	65.00 ± 50.74	45.67 ± 3.53	*F* = 0.14, *p* = 0.723
***C. papaya***	**0 ± 0**	**5.67 ± 5.67**	**TS = 1.00, *p* ≤ 0.001**
*C. spinosa*	77.67 ± 6.64	55.33 ± 8.41	*F* = 4.34, *p* = 0.106
***E. cheiri***	**0.33 ± 0.33**	**58.67 ± 2.33**	**TS = 3.00, *p* ≤ 0.001**
***I. amara***	**2.67 ± 2.19**	**37.33 ± 8.21**	***F* = 16.64, *p* = 0.015**
***L. douglasii***	**0 ± 0**	**60.33 ± 6.77**	**TS = 1.00, *p* ≤ 0.001**
***M. oleifera***	**0 ± 0**	**4.33 ± 2.19**	**TS = 1.00, *p* ≤ 0.001**
***P. sativum***	**0 ± 0**	**1.00 ± 1.00**	**TS = 1.00, *p* ≤ 0.001**
***R. odorata***	**12.33 ± 1.45**	**3.00 ± 3.00**	**TS = 3.00, *p* ≤ 0.001**
*T. majus*	17.00 ± 16.34	16.33 ± 14.38	TS = 6.00, *p* = 0.857

**Table 4:** Two-choice preference between abaxial and adaxial leaf surfaces shown as the percentage of eggs laid abaxially (mean ± SE) in each of the tested plants in *P. rapae* and *P. xylostella* larvae reared on cabbage. Data on the differences between the percentages of eggs laid abaxially and adaxially were analyzed using a one-tailed, two-sample test of proportions (*p* ≤ 0.05). The test compared the percentages of eggs laid abaxially and adaxially for each plant species. Significant *p*-values are shown in bold type. Values for *P. xylostella* taken from Badenes-Pérez et al. [38].

	% Abaxial Oviposition as Mean ± SE, n, Test Statistic, and *p*-Value	
	*P. rapae*	*P. xylostella*
*A. argenteum*	n/a	4.87 ± 2.23, n = 6; *z* = 3.12, *p* ≤ 0.001 *
*A. thaliana*	13.50 ± 2.02, n = 48; *z* = 7.15, *p* ≤ 0.001 *	53.41 ± 1.66, n = 96; *z* = 0.94, *p* = 0.172
*A. caucasica*	58.89 ± 21.63, n = 4; *z* = 0.50, *p* = 0.307	60.83 ± 4.67, n = 6; *z* = 0.76, *p* = 0.223
*B. vulgaris*	31.16 ± 4.19, n = 6; *z* = 1.30, *p* = 0.096	50.38 ± 11.71, n = 6; *z* = 0.00, *p* = 0.500
*B. oleracea*	69.33 ± 7.78, n = 6; *z* = 1.34, *p* = 0.090	32.90 ± 11.86, n = 6; *z* = 1.18, *p* = 0.119
*B. orientalis*	52.69 ± 8.47, n = 6; *z* = 0.19, *p* = 0.426	44.50 ± 9.38, n = 6; *z* = 0.42, *p* = 0.339
*C. bursa-pastoris*	n/a	40.71 ± 16.36, n = 5; *z* = 0.57, *p* = 0.285
*C. pratensis*	40.20 ± 14.57, n = 6; *z* = 0.68, *p* = 0.249	47.50 ± 4.61, n = 6; *z* = 0.14, *p* = 0.445
*C. papaya*	n/a	n/a
*C. spinosa*	26.15 ± 6.98, n = 5; *z* = 1.51, *p* = 0.066	33.46 ± 6.85, n = 5; *z* = 1.08, *p* = 0.141
*E. cheiri*	66.67 ± 30.33, n = 3; *z* = 0.82, *p* = 0.207	69.29 ± 2.96, n = 6; *z* = 1.20, *p* = 0.115
*I. amara*	15.48 ± 8.99, n = 4; *z* = 1.95, *p* = 0.025 *	22.12 ± 4.74, n = 6; *z* = 1.94, *p* = 0.020 *
*L. douglasii*	n/a	24.15 ± 1.33, n = 6; *z* = 1.80, *p* = 0.036 *
*M. oleifera*	n/a	n/a
*P. sativum*	n/a	n/a
*R. odorata*	68.90 ± 9.63, n = 6; *z* = 1.31, *p* = 0.095	48.33 ± 25.87, n = 3; *z* = 0.10, *p* = 0.461
*T. majus*	n/a	75.93 ± 14.46, n = 3; *z* = 1.27, *p* = 0.100

* Adaxial leaf surface preferred. n/a: not available, not possible to calculate because oviposition occurred in less than three replicates.

**Table 5:** Survival from first-instar larvae to pupae (mean ± SE) for *P. rapae* and *P. xylostella* larvae reared on cabbage. Data comparing survival of larvae of these two species were analyzed using a one-tailed, two-sample test of proportions (*p* ≤ 0.05) (n = 3, except for *P. xylostella* on *A. argenteum*, *A. caucasica*, *E. cheiri*, *I. amara*, *M. oleifera*, and *R. odorata* in which n = 4 and on *T. majus* in which n = 5). Significant *p*-values are shown in bold type. Larval survival values for *P. xylostella* taken from Badenes-Pérez et al. [37].

	Survival of Larvae (%) per Plant		
	*P. rapae*	*P. xylostella*	Test Statistic and *p*-Value
*A. argenteum*	0 ± 0	20.0 ± 8.2	*z* = 0.82, *p* = 0.205
*A. thaliana*	53.3 ± 6.7	46.7 ± 17.6	*z* = 0.71, *p* = 0.239
*A. caucasica*	0 ± 0	25.0 ± 18.9	*z* = 0.94, *p* = 0.175
*B. vulgaris*	26.7 ± 6.7	0 ± 0	*z* = 0.96, *p* = 0.168
*B. oleracea*	93.3 ± 6.7	33.3 ± 6.7	*z* = 1.52, *p* = 0.064
*B. orientalis*	0 ± 0	13.3 ± 6.7	*z* = 0.65, *p* = 0.256
*C. bursa-pastoris*	0 ± 0	20.0 ± 11.5	*z* = 0.82, *p* = 0.207
*C. pratensis*	93.3 ± 6.7	66.7 ± 6.7	*z* = 0.82, *p* = 0.207
*C. papaya*	0 ± 0	0 ± 0	*z* = 0.0, *p* = 0.5
*C. spinosa*	0 ± 0	6.7 ± 6.7	*z* = 0.45, *p* = 0.325
*E. cheiri*	0 ± 0	50.0 ± 12.9	*z* = 1.45, *p* = 0.074
*I. amara*	0 ± 0	40.0 ± 14.1	*z* = 1.25, *p* = 0.106
*L. douglasii*	0 ± 0	66.7 ± 6.7	***z* = 1.73, *p* = 0.042**
*M. oleifera*	0 ± 0	10.0 ± 10.0	*z* = 0.56, *p* = 0.287
*P. sativum*	0 ± 0	0 ± 0	*z* = 0.0, *p* = 0.5
*R. odorata*	80.0 ± 20.0	20.0 ± 20.0	*z* = 1.58, *p* = 0.057
*T. majus*	0 ± 0	24.0 ± 14.7	*z* = 0.92, *p* = 0.179

**Table 6:** Plants used in the experiments.

Family	Subfamily	Species	Common Name
Brassicaceae	Alysseae	*Alyssum argenteum* All.	Yellow tuft
Brassicaceae	Arabideae	*Arabis caucasica* Willd.	Mountain rock cress
Brassicaceae	Brassiceae	*Brassica oleracea* var. *capitata* L.	Cabbage
Brassicaceae	Camelineae	*Arabidopsis thaliana* (L.) Heynh.	Thale cress
Brassicaceae	Camelineae	*Capsella bursa-pastoris* (L.) Medik.	Shepherd’s purse
Brassicaceae	Camelineae	*Erysimum cheiri* (L.) Crantz	Wallflower
Brassicaceae	Cardamineae	*Barbarea vulgaris* R.Br.	Wintercress
Brassicaceae	Cardamineae	*Cardamine pratensis* L.	Cuckoo flower
Brassicaceae	Euclidieae	*Bunias orientalis* L.	Turkish rocket
Brassicaceae	Iberideae	*Iberis amara* L.	Bitter candytuft
Caricaceae	-	*Carica papaya* L.	Papaya
Cleomaceae	-	*Cleome spinosa* L.	Spider flower
Fabaceae	-	*Pisum sativum* L.	Pea
Limnanthaceae	-	*Limnanthes douglasii* R. Br.	Douglas’ meadowfoam
Moringaceae	-	*Moringa oleifera* Lam.	Drumstick tree
Resedaceae	-	*Reseda odorata* L.	Common mignonette
Tropaeolaceae	-	*Tropaeolum majus* L.	Garden nasturtium

## Data Availability

The data that support the findings of this study are available from the author upon reasonable request.

## Supplementary Materials

The following supporting information can be downloaded at https://www.mdpi.com/article/10.3390/plants12112148/s1, Table S1: Pairwise comparisons in OPI between plant species after conducting Kruskal–Wallis tests; Table S2: Comparison between *P. rapae* and *P. xylostella* for the percentage of eggs laid on plant species tested compared to *A. thaliana* (n = 3). Significant differences are shown in bold type; Table S3: Significance of correlations between oviposition preference index in two-choice tests (OPI), total oviposition in no-choice tests (TO), and larval survival (LS) for *P. rapae* and *P. xylostella* in the plants tested; Table S4: Mean ± SE glucosinolate content (µmol g-1 plant dry weight) in the plants tested [37]; Table S5: Total glucosinolate content (TOT) and content of aliphatic glucosinolates with sulfur-containing side chains (AS), other aliphatic glucosinolates (AO), benzenic glucosinolates (BEN), and indolic glucosinolates (IN) for each of the plant types tested (A). Glucosinolate richness (S), Shannon’s diversity index for the four glucosinolate classes (HA), Shannon’s diversity index for the relative concentrations of all individual glucosinolates (HB), and chemical complexity index for glucosinolates (CCI) for each of the plant types tested (B) [37]; Table S6: Correlations between oviposition preference index (OPI), total oviposition (TO), and larval survival (LS) and glucosinolate richness (S), Shannon’s diversity index for the four glucosinolate classes (HA), Shannon’s diversity index for the relative concentrations of all individual glucosinolates (HB), glucosinolate complexity index (GCI), total glucosinolate content (TOT), aliphatic glucosinolates with sulfur-containing side chains (AS), other aliphatic glucosinolates (AO), benzenic (BEN), and indolic glucosinolates (IN) as shown by CATPCA analysis; Table S7: Pairwise comparisons in total oviposition in no-choice tests (TO) between plant species after conducting Kruskal–Wallis tests; Table S8: Comparison between *P. rapae* and *P. xylostella* for the percentage of eggs laid on the abaxial side of the leaves in the plant species tested (n = 3–96, except in the case of *A. argenteum*, *C. bursa-pastoris*, and *T. majus* for *P. rapae* and in the case of *C. papaya* and *M. oleifera* for *P. xylostella*, in which n = 2); Table S9: Pairwise comparisons in total oviposition in no-choice tests (TO) between plant species after conducting Kruskal–Wallis tests; Table S8: Pairwise comparisons in larval survival (LS) between plant species after conducting Kruskal–Wallis tests; Table S10: Origin of the seeds of the plant species tested; Figure S1: CATPCA plots showing the relationship between oviposition preference index (OPI) (A), total oviposition (TO) (B), and larval survival (LS) (C) and total glucosinolate content (TOT) and content of aliphatic glucosinolates with sulfur-containing side chains (AS), other aliphatic glucosinolates (AO), benzenic glucosinolates (BEN), and indolic glucosinolates (IN) in the plant species tested. Reference [37] is cited in the Supplementary Materials.
