# Supplementary material for: Plant Glucosinolate Content and Host-Plant Preference and Suitability in the Small White Butterfly (Lepidoptera: Pieridae) and Comparison with Another Specialist Lepidopteran
Source: Plants (Basel). 2023 May 29;12(11):2148. doi: 10.3390/plants12112148 (PMC10255426; doi:10.3390/plants12112148)
Supplement: Supplementary file 1 [file plants-12-02148-s001.zip › plants-2317911-supplementary.pdf]

Plant glucosinolate content and host-plant preference and suitability in the small white butterfly (Lepidoptera: Pieridae) and comparison with another specialist lepidopteran

Francisco R. Badenes-Perez[1, 2]

[1] *Max Planck Institute for Chemical Ecology, 07745 Jena, Germany*

[2] *Instituto de Ciencias Agrarias, Consejo Superior de Investigaciones Científicas, Madrid, 28006, Spain*

Table S1. Pairwise comparisons in OPI between plant species after conducting Kruskal-Wallis test. The test statistic (TS) and *p*-values of the Kruskal-Wallis tests were TS=35.63 and *p*=0.002 and TS=35.44 and *p*=0.002 for *P. rapae* and *P. xylostella*, respectively. The pairwise comparisons among plant species is shown below for *P. rapae* (A) and *P. xylostella* (B). Each row tests the null hypothesis that the Sample 1 and Sample 2 distributions are the same. Asymptotic significances (2-sided tests) are displayed. The significance level is 0.050.

A

Pairwise comparisons of plant species for *P. rapae* OPI

Sample 1-Sample 2	Test Statistic	Std. Error	Std. Test Statistic	Sig.
M. oleifera-A. argenteum	4.167	10.867	.383	.701
L. douglasii-R. odorata	-29.500	10.867	-2.715	.007
M. oleifera-B. vulgaris	29.833	10.867	2.745	.006
L. douglasii-C. bursa-pastoris	5.167	10.867	.475	.634
L. douglasii-B. orientalis	34.500	10.867	3.175	.001
M. oleifera-C. papaya	5.500	10.867	.506	.613
M. oleifera-B. orientalis	34.500	10.867	3.175	.001
T. majus-C. papaya	5.500	10.867	.506	.613
L. douglasii-A. argenteum	4.167	10.867	.383	.701
L. douglasii-C. pratensis	25.500	10.867	2.347	.019
T. majus-B. orientalis	34.500	10.867	3.175	.001
M. oleifera-C. pratensis	25.500	10.867	2.347	.019
T. majus-A. argenteum	4.167	10.867	.383	.701
T. majus-iberis	-12.833	10.867	-1.181	.238
T. majus-C. pratensis	25.500	10.867	2.347	.019
L. douglasii-C. spinosa	16.000	10.867	1.472	.141
M. oleifera-C. spinosa	16.000	10.867	1.472	.141
T. majus-C. spinosa	16.000	10.867	1.472	.141
L. douglasii-I. amara	10.667	10.867	.982	.326
M. oleifera-I. amara	10.667	10.867	.982	.326
T. majus-I. amara	10.667	10.867	.982	.326
L. douglasii-M. oleifera	.000	10.867	.000	1.000
T. majus-B. vulgaris	29.833	10.867	2.745	.006

L. douglasii-A. caucasica	6.667	10.867	.613	.540
M. oleifera-A. caucasica	6.667	10.867	.613	.540
L. douglasii-T. majus	.000	10.867	.000	1.000
L. douglasii-P. sativum	-4.500	10.867	-.414	.679
T. majus-A. caucasica	6.667	10.867	.613	.540
L. douglasii-C. papaya	5.500	10.867	.506	.613
L. douglasii-iberis	-12.833	10.867	-1.181	.238
L. douglasii-B. vulgaris	29.833	10.867	2.745	.006
M. oleifera-T. majus	.000	10.867	.000	1.000
M. oleifera-P. sativum	-4.500	10.867	-.414	.679
M. oleifera-C. bursa-pastoris	5.167	10.867	.475	.634
M. oleifera-R. odorata	-29.500	10.867	-2.715	.007
T. majus-C. bursa-pastoris	5.167	10.867	.475	.634
L. douglasii-B. oleracea	23.167	10.867	2.132	.033
M. oleifera-iberis	-12.833	10.867	-1.181	.238
M. oleifera-B. oleracea	23.167	10.867	2.132	.033
T. majus-P. sativum	4.500	10.867	.414	.679
T. majus-B. oleracea	23.167	10.867	2.132	.033
T. majus-R. odorata	29.500	10.867	2.715	.007
A. argenteum-P. sativum	-.333	10.867	-.031	.976
A. argenteum-C. bursa-pastoris	-1.000	10.867	-.092	.927
A. argenteum-C. papaya	-1.333	10.867	-.123	.902
A. argenteum-A. caucasica	-2.500	10.867	-.230	.818
A. argenteum-I. amara	-6.500	10.867	-.598	.550
A. argenteum-iberis	-8.667	10.867	-.798	.425
A. argenteum-C. spinosa	-11.833	10.867	-1.089	.276
A. argenteum-B. oleracea	-19.000	10.867	-1.748	.080
A. argenteum-C. pratensis	-21.333	10.867	-1.963	.050
A. argenteum-R. odorata	-25.333	10.867	-2.331	.020
A. argenteum-B. vulgaris	-25.667	10.867	-2.362	.018
A. argenteum-B. orientalis	-30.333	10.867	-2.791	.005
P. sativum-C. bursa-pastoris	.667	10.867	.061	.951
P. sativum-C. papaya	1.000	10.867	.092	.927
P. sativum-A. caucasica	2.167	10.867	.199	.842
P. sativum-I. amara	6.167	10.867	.567	.570
P. sativum-iberis	-8.333	10.867	-.767	.443
P. sativum-C. spinosa	11.500	10.867	1.058	.290
P. sativum-B. oleracea	18.667	10.867	1.718	.086

P. sativum-C. pratensis	21.000	10.867	1.932	.053
P. sativum-R. odorata	-25.000	10.867	-2.301	.021
P. sativum-B. vulgaris	25.333	10.867	2.331	.020
P. sativum-B. orientalis	30.000	10.867	2.761	.006
C. bursa-pastoris-C. papaya	-.333	10.867	-.031	.976
C. bursa-pastoris-A. caucasica	1.500	10.867	.138	.890
C. bursa-pastoris-I. amara	-5.500	10.867	-.506	.613
C. bursa-pastoris-iberis	-7.667	10.867	-.705	.481
C. bursa-pastoris-C. spinosa	-10.833	10.867	-.997	.319
C. bursa-pastoris-B. oleracea	18.000	10.867	1.656	.098
C. bursa-pastoris-C. pratensis	-20.333	10.867	-1.871	.061
C. bursa-pastoris-R. odorata	-24.333	10.867	-2.239	.025
C. bursa-pastoris-B. vulgaris	24.667	10.867	2.270	.023
C. bursa-pastoris-B. orientalis	29.333	10.867	2.699	.007
C. papaya-A. caucasica	1.167	10.867	.107	.915
C. papaya-I. amara	-5.167	10.867	-.475	.634
C. papaya-iberis	-7.333	10.867	-.675	.500
C. papaya-C. spinosa	-10.500	10.867	-.966	.334
C. papaya-B. oleracea	17.667	10.867	1.626	.104
C. papaya-C. pratensis	-20.000	10.867	-1.840	.066
C. papaya-R. odorata	-24.000	10.867	-2.209	.027
C. papaya-B. vulgaris	24.333	10.867	2.239	.025
C. papaya-B. orientalis	29.000	10.867	2.669	.008
A. caucasica-I. amara	-4.000	10.867	-.368	.713
A. caucasica-iberis	-6.167	10.867	-.567	.570
A. caucasica-C. spinosa	-9.333	10.867	-.859	.390
A. caucasica-B. oleracea	-16.500	10.867	-1.518	.129
A. caucasica-C. pratensis	-18.833	10.867	-1.733	.083
A. caucasica-R. odorata	-22.833	10.867	-2.101	.036
A. caucasica-B. vulgaris	-23.167	10.867	-2.132	.033
A. caucasica-B. orientalis	-27.833	10.867	-2.561	.010
I. amara-iberis	-2.167	10.867	-.199	.842
I. amara-C. spinosa	5.333	10.867	.491	.624
I. amara-B. oleracea	12.500	10.867	1.150	.250
I. amara-C. pratensis	14.833	10.867	1.365	.172
I. amara-R. odorata	-18.833	10.867	-1.733	.083
I. amara-B. vulgaris	19.167	10.867	1.764	.078
I. amara-B. orientalis	23.833	10.867	2.193	.028

iberis-C. spinosa	3.167	10.867	.291	.771
iberis-B. oleracea	10.333	10.867	.951	.342
iberis-C. pratensis	12.667	10.867	1.166	.244
iberis-R. odorata	16.667	10.867	1.534	.125
iberis-B. vulgaris	17.000	10.867	1.564	.118
iberis-B. orientalis	21.667	10.867	1.994	.046
C. spinosa-B. oleracea	7.167	10.867	.659	.510
C. spinosa-C. pratensis	9.500	10.867	.874	.382
C. spinosa-R. odorata	-13.500	10.867	-1.242	.214
C. spinosa-B. vulgaris	13.833	10.867	1.273	.203
C. spinosa-B. orientalis	18.500	10.867	1.702	.089
B. oleracea-C. pratensis	-2.333	10.867	-.215	.830
B. oleracea-R. odorata	-6.333	10.867	-.583	.560
B. oleracea-B. vulgaris	-6.667	10.867	-.613	.540
B. oleracea-B. orientalis	-11.333	10.867	-1.043	.297
C. pratensis-R. odorata	-4.000	10.867	-.368	.713
C. pratensis-B. vulgaris	4.333	10.867	.399	.690
C. pratensis-B. orientalis	9.000	10.867	.828	.408
R. odorata-B. vulgaris	.333	10.867	.031	.976
R. odorata-B. orientalis	5.000	10.867	.460	.645
B. vulgaris-B. orientalis	4.667	10.867	.429	.668

B

Pairwise comparisons of plant species for *P. xylostella* OPI

Sample 1-Sample 2	Test Statistic	Std. Error	Std. Test Statistic	Sig.
P. sativum-T. majus	-5.500	11.289	-.487	.626
P. sativum-A. argenteum	11.833	11.289	1.048	.295
P. sativum-R. odorata	-17.000	11.289	-1.506	.132
M. oleifera-T. majus	-5.500	11.289	-.487	.626
M. oleifera-R. odorata	-17.000	11.289	-1.506	.132
P. sativum-C. bursa-pastoris	7.000	11.289	.620	.535
M. oleifera-P. sativum	.000	11.289	.000	1.000
M. oleifera-B. orientalis	17.500	11.289	1.550	.121
P. sativum-L. douglasii	38.833	11.289	3.440	<.001
M. oleifera-L. douglasii	38.833	11.289	3.440	<.001

P. sativum-C. papaya	6.500	11.289	.576	.565
M. oleifera-C. papaya	6.500	11.289	.576	.565
M. oleifera-C. bursa-pastoris	7.000	11.289	.620	.535
M. oleifera-A. argenteum	11.833	11.289	1.048	.295
M. oleifera-B. oleracea	22.167	11.289	1.964	.050
M. oleifera-I. amara	26.500	11.289	2.347	.019
M. oleifera-B. vulgaris	36.833	11.289	3.263	.001
P. sativum-E. cheiri	14.333	11.289	1.270	.204
M. oleifera-E. cheiri	14.333	11.289	1.270	.204
P. sativum-C. spinosa	10.667	11.289	.945	.345
P. sativum-B. orientalis	17.500	11.289	1.550	.121
P. sativum-B. oleracea	22.167	11.289	1.964	.050
M. oleifera-C. spinosa	10.667	11.289	.945	.345
P. sativum-I. amara	26.500	11.289	2.347	.019
M. oleifera-A. caucasica	27.167	11.289	2.406	.016
P. sativum-A. caucasica	27.167	11.289	2.406	.016
P. sativum-C. pratensis	30.167	11.289	2.672	.008
P. sativum-B. vulgaris	36.833	11.289	3.263	.001
M. oleifera-C. pratensis	30.167	11.289	2.672	.008
T. majus-C. papaya	1.000	11.289	.089	.929
T. majus-C. bursa-pastoris	1.500	11.289	.133	.894
T. majus-C. spinosa	5.167	11.289	.458	.647
T. majus-A. argenteum	6.333	11.289	.561	.575
T. majus-E. cheiri	8.833	11.289	.782	.434
T. majus-R. odorata	11.500	11.289	1.019	.308
T. majus-B. orientalis	12.000	11.289	1.063	.288
T. majus-B. oleracea	16.667	11.289	1.476	.140
T. majus-I. amara	21.000	11.289	1.860	.063
T. majus-A. caucasica	21.667	11.289	1.919	.055
T. majus-C. pratensis	24.667	11.289	2.185	.029
T. majus-B. vulgaris	31.333	11.289	2.776	.006
T. majus-L. douglasii	33.333	11.289	2.953	.003
C. papaya-C. bursa-pastoris	.500	11.289	.044	.965
C. papaya-C. spinosa	-4.167	11.289	-.369	.712
C. papaya-A. argenteum	5.333	11.289	.472	.637
C. papaya-E. cheiri	-7.833	11.289	-.694	.488
C. papaya-R. odorata	-10.500	11.289	-.930	.352
C. papaya-B. orientalis	11.000	11.289	.974	.330

C. papaya-B. oleracea	15.667	11.289	1.388	.165
C. papaya-I. amara	-20.000	11.289	-1.772	.076
C. papaya-A. caucasica	20.667	11.289	1.831	.067
C. papaya-C. pratensis	-23.667	11.289	-2.096	.036
C. papaya-B. vulgaris	30.333	11.289	2.687	.007
C. papaya-L. douglasii	-32.333	11.289	-2.864	.004
C. bursa-pastoris-C. spinosa	-3.667	11.289	-.325	.745
C. bursa-pastoris-A. argenteum	4.833	11.289	.428	.669
C. bursa-pastoris-E. cheiri	-7.333	11.289	-.650	.516
C. bursa-pastoris-R. odorata	-10.000	11.289	-.886	.376
C. bursa-pastoris-B. orientalis	10.500	11.289	.930	.352
C. bursa-pastoris-B. oleracea	15.167	11.289	1.344	.179
C. bursa-pastoris-I. amara	-19.500	11.289	-1.727	.084
C. bursa-pastoris-A. caucasica	20.167	11.289	1.786	.074
C. bursa-pastoris-C. pratensis	-23.167	11.289	-2.052	.040
C. bursa-pastoris-B. vulgaris	29.833	11.289	2.643	.008
C. bursa-pastoris-L. douglasii	-31.833	11.289	-2.820	.005
C. spinosa-A. argenteum	1.167	11.289	.103	.918
C. spinosa-E. cheiri	-3.667	11.289	-.325	.745
C. spinosa-R. odorata	-6.333	11.289	-.561	.575
C. spinosa-B. orientalis	6.833	11.289	.605	.545
C. spinosa-B. oleracea	11.500	11.289	1.019	.308
C. spinosa-I. amara	-15.833	11.289	-1.403	.161
C. spinosa-A. caucasica	16.500	11.289	1.462	.144
C. spinosa-C. pratensis	19.500	11.289	1.727	.084
C. spinosa-B. vulgaris	26.167	11.289	2.318	.020
C. spinosa-L. douglasii	-28.167	11.289	-2.495	.013
A. argenteum-E. cheiri	-2.500	11.289	-.221	.825
A. argenteum-R. odorata	-5.167	11.289	-.458	.647
A. argenteum-B. orientalis	-5.667	11.289	-.502	.616
A. argenteum-B. oleracea	-10.333	11.289	-.915	.360
A. argenteum-I. amara	-14.667	11.289	-1.299	.194
A. argenteum-A. caucasica	-15.333	11.289	-1.358	.174
A. argenteum-C. pratensis	-18.333	11.289	-1.624	.104
A. argenteum-B. vulgaris	-25.000	11.289	-2.215	.027
A. argenteum-L. douglasii	-27.000	11.289	-2.392	.017
E. cheiri-R. odorata	-2.667	11.289	-.236	.813
E. cheiri-B. orientalis	3.167	11.289	.281	.779

E. cheiri-B. oleracea	7.833	11.289	.694	.488
E. cheiri-I. amara	-12.167	11.289	-1.078	.281
E. cheiri-A. caucasica	12.833	11.289	1.137	.256
E. cheiri-C. pratensis	15.833	11.289	1.403	.161
E. cheiri-B. vulgaris	22.500	11.289	1.993	.046
E. cheiri-L. douglasii	-24.500	11.289	-2.170	.030
R. odorata-B. orientalis	.500	11.289	.044	.965
R. odorata-B. oleracea	5.167	11.289	.458	.647
R. odorata-I. amara	9.500	11.289	.842	.400
R. odorata-A. caucasica	10.167	11.289	.901	.368
R. odorata-C. pratensis	13.167	11.289	1.166	.243
R. odorata-B. vulgaris	19.833	11.289	1.757	.079
R. odorata-L. douglasii	21.833	11.289	1.934	.053
B. orientalis-B. oleracea	4.667	11.289	.413	.679
B. orientalis-I. amara	-9.000	11.289	-.797	.425
B. orientalis-A. caucasica	9.667	11.289	.856	.392
B. orientalis-C. pratensis	-12.667	11.289	-1.122	.262
B. orientalis-B. vulgaris	-19.333	11.289	-1.713	.087
B. orientalis-L. douglasii	-21.333	11.289	-1.890	.059
B. oleracea-I. amara	-4.333	11.289	-.384	.701
B. oleracea-A. caucasica	5.000	11.289	.443	.658
B. oleracea-C. pratensis	-8.000	11.289	-.709	.479
B. oleracea-B. vulgaris	-14.667	11.289	-1.299	.194
B. oleracea-L. douglasii	-16.667	11.289	-1.476	.140
I. amara-A. caucasica	.667	11.289	.059	.953
I. amara-C. pratensis	3.667	11.289	.325	.745
I. amara-B. vulgaris	10.333	11.289	.915	.360
I. amara-L. douglasii	-12.333	11.289	-1.093	.275
A. caucasica-C. pratensis	-3.000	11.289	-.266	.790
A. caucasica-B. vulgaris	-9.667	11.289	-.856	.392
A. caucasica-L. douglasii	-11.667	11.289	-1.033	.301
C. pratensis-B. vulgaris	6.667	11.289	.591	.555
C. pratensis-L. douglasii	-8.667	11.289	-.768	.443
B. vulgaris-L. douglasii	-2.000	11.289	-.177	.859

Table S2. Comparison betweeen *P. rapae* and *P. xylostella* for the percentage of eggs laid on plant species tested compared to *A. thaliana* (n=3). Significant differences are shown in bold type.

	Test Statistic, *p*-value
A. argenteum	*z*=0.42, *p*=0.336
A. caucasica	*z*=0.86, *p*=0.196
B. vulgaris	*z*=0.82, *p*=0.206
B. oleracea	*z*=0.09, *p*=0.463
B. orientalis	*z*=0.97, *p*=0.167
C. bursa-pastoris	*z*=0.14, *p*=0.445
C. pratensis	*z*=0.53, *p*=0.297
C. papaya	*z*=0.16, *p*=0.436
C. spinosa	*z*=0.39, *p*=0.350
E. cheiri	*z*=0.50, *p*=0.310
I. amara	*z*=0.84, *p*=0.201
L. douglasii	***z*=1.95, *p*=0.026***
M. oleifera	*z*=0.00, *p*=0.050
P. sativum	*z*=0.18, *p*=0.430
R. odorata	*z*=0.31, *p*=0.379
T. majus	*z*=0.43, *p*=0.333

Table S3. Significance of correlations between oviposition preference index in two-choice tests (OPI), total oviposition in no-choice tests (TO), and larval survival (LS) for *P. rapae* and *P. xylostella* in the plants tested. Two-tailed Spearman´s rho correlations were performed. Replication was n = 17 for the correlations between LS in *P. rapae* and LS in *P. xylostella* and n=16 for the correlations involving OPI and TO (*A. thaliana* was not included in the OPI and TO tests). Significant *p*-values ($p \leq 0.05$) are shown in bold type.

A

	P-value and correlation coefficient of Spearman´s rho correlation				
	TO *P. xylostella*	TO *P. rapae*	LS *P. xylostella*	LS *P. rapae*	OPI *P. xylostella*
OPI *P. rapae*	-	***P*≤0.001; 0.765**	-	*P*=0.059; 0.482	*P*=0.126; 0.398
OPI *P. xylostella*	***P*=0.027; 0.550**	-	***P*=0.035; 0.529**	-	-

B

	P-value and correlation coefficient of Spearman´s rho correlation	
	LS *P. xylostella*	TO *P. xylostella*	TO *P. rapae*
LS *P. rapae*	*P*=0.170; 0.348	-	***P*=0.018; 0.581**
LS *P. xylostella*	-	***P*=0.049; 0.500**	-

Table S4. Mean ± SE glucosinolate content (μmol g^{-1} plant dry weight) in the plants tested. From the total glucosinolate content the percentage of individual glucosinolates and the percentage of glucosinolates according to chemical class is shown. Four glucosinolate classes were considered: aliphatic with sulfur-containing side chains (AS), other aliphatic (AO), benzenic (BEN), and indolic (IN). Values based on means across replicates (n=3-26) taken from Badenes-Pérez et al. [37].

Plant species	Total glucosinolates (% AO, BEN, IN, and AS)	Identity of glucosinolates identified (% of total glucosinolates)
A. argenteum	1.4 ± 0.3 (0%, 0%, 75.32%, 24.68%)	5MSOP (75.32%), I3M (18.94%), 4OHI3M (4.33%), 4MOI3M (1.41%)
A. thaliana	17.6 ± 0.4 (0%, 0%, 14.81%, 85.19%)	4MSOB (65.00%), I3M (11.10%), 3MSOP (9.56%), 8MSOO (5.15%), 4MTB (4.50%), 4MOI3M (2.22%), 1MOI3M (1.23%), 7MSOH (0.97%), 4OHI3M (0.26%)
A. caucasica	20.8 ± 18.2 (60.03%, 0%, 0.78%, 39.19%)	1ME (53.43%), 9MSON (32.70%), 1MP (4.48%), 10MSOD (3.19%), 2MP (2.12%), 8MSOO (1.77%), 4MTB (1.53%), 4MOI3M (0.52%), 4OHI3M (0.26%)
B. vulgaris	35.6 ± 4.2 (0%, 81.60%, 18.40%, 0%)	S2OH2PE (80.88%), I3M (17.26%), 4MOI3M (0.80%), R2OH2PE (0.72%), 4OHI3M (0.34%)
B oleracea capitata	8.0 ± 1.5 (25.13%, 0%, 59.04%, 15.83%)	I3M (52.66%), A (24.13%), 3MSOP (15.06%), 4MOI3M (3.88%), 1MOI3M (1.72%), R2OH3B (1.00%), 4OHI3M (0.78%), 4MSOB (0.77%)
B. orientalis	33.3 ± 2.9 (0%, 99.66%, 0.34%, 0%)	4OHB (96.47%), 4MOHB (3.19%), 4MOI3M (0.23%), 4OHI3M (0.06%), I3M (0.05%)
C. bursa-pastoris	0 ± 0 (0%, 0%, 0%, 0%)	-
C. pratensis	27.1 ± 7.6 (95.10%, 0%, 4.90%, 0%)	3OHMP (93.92%), I3M (4.51%), 3MP (1.18%), 4OHI3M (0.39%)
C. papaya	4.1 ± 1.3 (0%, 99.72%, 0.28%, 0%)	B (99.72%), I3M (0.28%)
C. spinosa	39.9 ± 6.1 (97.44%, 0%, 2.56%, 0%)	M (97.44%), I3M (1.64%), 4OHI3M (0.84%), 4MOI3M (0.08%)
E. cheiri	16.3 ± 6.5 (0%, 0%, 0%, 100.00%)	3MSOOP (59.35%), 3MTP (25.10%), 3MSOP (14.98%), 4MSOB (0.57%)
I. amara	53.8 ± 9.8 (0%, 0%, 0.06%, 99.94%)	3MSOP (85.71%), 3MTP (13.85%), 4MSOB (0.37%), 4MOI3M (0.06%)
L. douglasii	49.4 ± 10.2 (0%, 99.97%, 0.03%, 0%)	3MOHB (93.61%), 3OHB (6.36%), 4OHI3M (0.02%), 1MOI3M (0.01%)
M. oleifera	28.0 ± 2.5 (0%, 100.00%, 0%, 0%)	4RB (87.94%), 4OHB (10.52%), B (1.54%)
P. sativum	0 ± 0 (0%, 0%, 0%, 0%)	-
R. odorata	89.8 ± 18.0 (0%, 93.88%, 6.12%, 0%)	2RB (92.96%), I3M (6.12%), 2AB (0.91%)
T. majus	28.0 ± 12.4 (0%, 100.00%, 0%, 0%)	B (99.66%), 4MOHB (0.34%)

Glucosinolate abbreviations were: Allyl (A), 2-Arabinobenzyl (2AB), Benzyl (B), 3-Hydroxybenzyl (3OHB), 4-Hydroxybenzyl (4OHB), 2(*R*)-Hydroxy-3-butenyl (R2OH3B), 3-(Hydroxymethyl)pentyl (3OHMP), 4-Hydroxyindol-3-ylmethyl (4OHI3M), 2(*R*)-Hydroxy-2-phenylethyl (R2OH2PE), 2(*S*)-Hydroxy-2-phenylethyl (S2OH2PE), Indol-3-ylmethyl (I3M), 3-Methoxybenzyl (3MOHB), 4-Methoxybenzyl (4MOHB), 1-Methoxyindol-3-ylmethyl (1MOI3M), 4-Methoxyindol-3-ylmethyl (4MOI3M), Methyl (M), 1-Methylethyl (1ME), 3Methylpentyl (3MP), 1-Methylpropyl (1MP), 2-Methylpropyl (2MP), 4-(Methylsulfinyl)butyl (4MSOB), 10-(Methylsulfinyl)decyl (10MSOD), 7-(Methylsulfinyl)heptyl (7MSOH), 9-(Methylsulfinyl)nonyl (9MSON), 8-(Methylsulfinyl)octyl (8MSOO), 5-(Methylsulfinyl)pentyl (5MSOP), 3-(Methylsulfinyl)propyl (3MSOP), 3-(Methylsulfonyl)propyl (3MSOOP), 4-(Methylthio)butyl (4MTB), 3-(Methylthio)propyl (3MTP), 2-(α-L-Rhamnopyranosyloxy)benzyl (2RB), 4-(α-L-Rhamnopyranosyloxy)benzyl (4RB).

Table S5. Total glucosinolate content (TOT) and content of aliphatic glucosinolates with sulfur-containing side chains (AS), other aliphatic glucosinolates (AO), benzenic glucosinolates (BEN), and indolic glucosinolates (IN) for each of the plant types tested (A). Glucosinolate richness (*S*), Shannon´s diversity index for the four glucosinolate classes (H_A), Shannon´s diversity index for the relative concentrations of all individual glucosinolates (H_B), and chemical complexity index for glucosinolates (CCI) for each of the plant types tested (B). Values based on means across replicates taken from Badenes-Pérez et al. [37].

A

	TOT	AS	AO	BEN	IN
A. argenteum	20.81	8.16	12.49	0.00	0.16
A. thaliana	97.95	0.12	93.74	0.00	4.09
A. caucasica	6.11	0.00	1.73	0.00	4.38
B. vulgaris	0.00	0.00	0.00	0.00	0.00
B. oleracea	30.72	27.54	1.31	0.00	0.18
B. orientalis	53.79	53.76	0.00	0.00	0.03
C. bursa-pastoris	120.51	0.06	0.00	120.44	0.00
C. pratensis	27.96	0.00	0.00	27.96	0.00
C. papaya	0.00	0.00	0.00	0.00	0.00
C. spinosa	89.83	0.00	0.00	84.33	5.50
E. cheiri	20.81	8.16	12.49	0.00	0.16
I. amara	97.95	0.12	93.74	0.00	4.09
L. douglasii	0.00	0.00	0.00	0.00	0.00
M. oleifera	4.12	0.00	0.00	4.11	0.01
P. sativum	37.41	33.78	1.56	0.00	0.17
R. odorata	16.34	16.34	0.00	0.00	0.00
T. majus	120.51	0.06	0.00	120.44	0.00

B

	S	H_A	H_B	$CCI=H_A+H_B$
A. argenteum	4	0.693	0.724	1.417
A. thaliana	9	0.693	1.240	1.933
A. caucasica	9	1.099	1.209	2.308
B. vulgaris	5	0.693	0.568	1.261
B. oleracea	8	1.099	1.283	2.382
B. orientalis	5	0.693	0.167	0.860
C. bursa-pastoris	0	n/a	n/a	0
C. pratensis	4	0.693	0.273	0.966
C. papaya	2	0.693	0.019	0.712
C. spinosa	4	0.693	0.139	0.832
E. cheiri	4	0.000	0.970	0.970
I. amara	4	0.693	0.431	1.124
L. douglasii	5	0.693	0.240	0.933
M. oleifera	3	0.000	0.414	0.414
P. sativum	0	n/a	n/a	0
R. odorata	3	0.693	0.282	0.975
T. majus	2	0.000	0.023	0.023

Table S6. Correlations between oviposition preference index (OPI), total oviposition (TO), and larval survival (LS) and glucosinolate richness (*S*), Shannon´s diversity index for the four glucosinolate classes (H_A), Shannon´s diversity index for the relative concentrations of all individual glucosinolates (H_B), glucosinolate complexity index (GCI), total glucosinolate content (TOT), aliphatic glucosinolates with sulfur-containing side chains (AS), other aliphatic glucosinolates (AO), benzenic (BEN), and indolic glucosinolates (IN) as shown by CATPCA analysis. Only values above 0.35 were considered for subsequent generalized linear model analysis.

	OPI	TO	LS
S	.199	.352	.289
H_A	.293	.454	.291
H_B	.128	.366	.318
GCI	.278	.493	.357
TOT	.455	.183	.143
AO	.194	.566	.141
BEN	.242	-.208	.021
IN	.409	.240	.415
AS	-.057	.162	.203
Insect[b]	.162	.378	.086

[b]Supplementary variable.

Table S7. Pairwise comparisons in total oviposition in no-choice tests (TO) between plant species after conducting Kruskal-Wallis test. The test statistic (TS) and *p*-values of the Kruskal-Wallis tests were TS=39.75 and $p \leq 0.001$ and TS=38.99 and $p \leq 0.001$ for *P. rapae* and *P. xylostella*, respectively. The pairwise comparisons among plant species is shown below for *P. rapae* (A) and *P. xylostella* (B). Each row tests the null hypothesis that the Sample 1 and Sample 2 distributions are the same. Asymptotic significances (2-sided tests) are displayed. The significance level is 0.050.

A

Pairwise comparisons of plant species for *P. rapae* TO

Sample 1-Sample 2	Test Statistic	Std. Error	Std. Test Statistic	Sig.
C. papaya-E. cheiri	-4.167	11.195	-.372	.710
C. papaya-C. bursa-pastoris	4.167	11.195	.372	.710
C. papaya-I. amara	-10.833	11.195	-.968	.333
C. papaya-B. orientalis	23.667	11.195	2.114	.035
L. douglasii-B. vulgaris	27.500	11.195	2.456	.014
C. papaya-A. argenteum	4.167	11.195	.372	.710
C. papaya-C. spinosa	-35.167	11.195	-3.141	.002
P. sativum-C. bursa-pastoris	4.167	11.195	.372	.710
P. sativum-B. vulgaris	27.500	11.195	2.456	.014
L. douglasii-A. argenteum	4.167	11.195	.372	.710
L. douglasii-B. orientalis	23.667	11.195	2.114	.035
M. oleifera-A. argenteum	4.167	11.195	.372	.710
P. sativum-A. argenteum	4.167	11.195	.372	.710
M. oleifera-B. orientalis	23.667	11.195	2.114	.035
P. sativum-B. orientalis	23.667	11.195	2.114	.035
C. papaya-C. pratensis	-28.000	11.195	-2.501	.012
M. oleifera-E. cheiri	4.167	11.195	.372	.710
L. douglasii-E. cheiri	4.167	11.195	.372	.710
M. oleifera-C. bursa-pastoris	4.167	11.195	.372	.710
C. papaya-A. caucasica	18.167	11.195	1.623	.105
P. sativum-C. spinosa	35.167	11.195	3.141	.002
M. oleifera-C. spinosa	35.167	11.195	3.141	.002
L. douglasii-C. bursa-pastoris	4.167	11.195	.372	.710
L. douglasii-A. caucasica	18.167	11.195	1.623	.105
L. douglasii-C. spinosa	35.167	11.195	3.141	.002

M. oleifera-A. caucasica	18.167	11.195	1.623	.105
P. sativum-A. caucasica	18.167	11.195	1.623	.105
P. sativum-C. pratensis	28.000	11.195	2.501	.012
M. oleifera-C. pratensis	28.000	11.195	2.501	.012
L. douglasii-C. pratensis	28.000	11.195	2.501	.012
C. papaya-T. majus	-12.000	10.472	-1.146	.252
M. oleifera-B. vulgaris	27.500	11.195	2.456	.014
C. papaya-P. sativum	.000	11.195	.000	1.000
C. papaya-M. oleifera	.000	11.195	.000	1.000
C. papaya-R. odorata	-23.500	11.195	-2.099	.036
C. papaya-B. vulgaris	27.500	11.195	2.456	.014
C. papaya-B. oleracea	33.333	11.195	2.978	.003
L. douglasii-B. oleracea	33.333	11.195	2.978	.003
M. oleifera-B. oleracea	33.333	11.195	2.978	.003
P. sativum-B. oleracea	33.333	11.195	2.978	.003
C. papaya-L. douglasii	.000	11.195	.000	1.000
P. sativum-E. cheiri	4.167	11.195	.372	.710
L. douglasii-I. amara	10.833	11.195	.968	.333
M. oleifera-I. amara	10.833	11.195	.968	.333
P. sativum-I. amara	10.833	11.195	.968	.333
L. douglasii-M. oleifera	.000	11.195	.000	1.000
L. douglasii-P. sativum	.000	11.195	.000	1.000
L. douglasii-T. majus	-12.000	10.472	-1.146	.252
L. douglasii-R. odorata	-23.500	11.195	-2.099	.036
M. oleifera-P. sativum	.000	11.195	.000	1.000
M. oleifera-T. majus	-12.000	10.472	-1.146	.252
M. oleifera-R. odorata	-23.500	11.195	-2.099	.036
P. sativum-T. majus	-12.000	10.472	-1.146	.252
P. sativum-R. odorata	-23.500	11.195	-2.099	.036
C. bursa-pastoris-B. oleracea	29.167	11.195	2.605	.009
E. cheiri-B. oleracea	29.167	11.195	2.605	.009
C. bursa-pastoris-B. orientalis	19.500	11.195	1.742	.082
E. cheiri-B. orientalis	19.500	11.195	1.742	.082
C. bursa-pastoris-B. vulgaris	23.333	11.195	2.084	.037
E. cheiri-T. majus	-7.833	10.472	-.748	.454
E. cheiri-B. vulgaris	23.333	11.195	2.084	.037
C. bursa-pastoris-E. cheiri	.000	11.195	.000	1.000
C. bursa-pastoris-I. amara	-6.667	11.195	-.596	.552

C. bursa-pastoris-T. majus	-7.833	10.472	-.748	.454
C. bursa-pastoris-R. odorata	-19.333	11.195	-1.727	.084
C. bursa-pastoris-C. pratensis	-23.833	11.195	-2.129	.033
C. bursa-pastoris-C. spinosa	-31.000	11.195	-2.769	.006
E. cheiri-I. amara	-6.667	11.195	-.596	.552
E. cheiri-C. pratensis	23.833	11.195	2.129	.033
E. cheiri-C. spinosa	31.000	11.195	2.769	.006
A. argenteum-A. caucasica	-14.000	11.195	-1.251	.211
E. cheiri-R. odorata	-19.333	11.195	-1.727	.084
A. argenteum-C. bursa-pastoris	.000	11.195	.000	1.000
A. argenteum-E. cheiri	.000	11.195	.000	1.000
A. argenteum-I. amara	-6.667	11.195	-.596	.552
A. argenteum-T. majus	-7.833	10.472	-.748	.454
A. argenteum-R. odorata	-19.333	11.195	-1.727	.084
A. argenteum-B. orientalis	-19.500	11.195	-1.742	.082
A. argenteum-B. vulgaris	-23.333	11.195	-2.084	.037
A. argenteum-C. pratensis	-23.833	11.195	-2.129	.033
A. argenteum-B. oleracea	-29.167	11.195	-2.605	.009
A. argenteum-C. spinosa	-31.000	11.195	-2.769	.006
C. bursa-pastoris-A. caucasica	14.000	11.195	1.251	.211
E. cheiri-A. caucasica	14.000	11.195	1.251	.211
I. amara-T. majus	-1.167	10.472	-.111	.911
I. amara-A. caucasica	7.333	11.195	.655	.512
I. amara-R. odorata	-12.667	11.195	-1.131	.258
I. amara-B. orientalis	12.833	11.195	1.146	.252
I. amara-B. vulgaris	16.667	11.195	1.489	.137
I. amara-C. pratensis	17.167	11.195	1.533	.125
I. amara-B. oleracea	22.500	11.195	2.010	.044
I. amara-C. spinosa	24.333	11.195	2.174	.030
T. majus-A. caucasica	6.167	10.472	.589	.556
T. majus-R. odorata	11.500	10.472	1.098	.272
T. majus-B. orientalis	11.667	10.472	1.114	.265
T. majus-B. vulgaris	15.500	10.472	1.480	.139
T. majus-C. pratensis	16.000	10.472	1.528	.127
T. majus-B. oleracea	21.333	10.472	2.037	.042
T. majus-C. spinosa	23.167	10.472	2.212	.027
A. caucasica-R. odorata	-5.333	11.195	-.476	.634
A. caucasica-B. orientalis	-5.500	11.195	-.491	.623

A. caucasica-B. vulgaris	-9.333	11.195	-.834	.404
A. caucasica-C. pratensis	-9.833	11.195	-.878	.380
A. caucasica-B. oleracea	-15.167	11.195	-1.355	.175
A. caucasica-C. spinosa	-17.000	11.195	-1.519	.129
R. odorata-B. orientalis	.167	11.195	.015	.988
R. odorata-B. vulgaris	4.000	11.195	.357	.721
R. odorata-C. pratensis	4.500	11.195	.402	.688
R. odorata-B. oleracea	9.833	11.195	.878	.380
R. odorata-C. spinosa	11.667	11.195	1.042	.297
B. orientalis-B. vulgaris	-3.833	11.195	-.342	.732
B. orientalis-C. pratensis	-4.333	11.195	-.387	.699
B. orientalis-B. oleracea	9.667	11.195	.863	.388
B. orientalis-C. spinosa	-11.500	11.195	-1.027	.304
B. vulgaris-C. pratensis	-.500	11.195	-.045	.964
B. vulgaris-B. oleracea	5.833	11.195	.521	.602
B. vulgaris-C. spinosa	-7.667	11.195	-.685	.493
C. pratensis-B. oleracea	5.333	11.195	.476	.634
C. pratensis-C. spinosa	-7.167	11.195	-.640	.522
B. oleracea-C. spinosa	-1.833	11.195	-.164	.870

B

Pairwise comparisons of plant species for *P. xylostella* TO

Sample 1-Sample 2	Test Statistic	Std. Error	Std. Test Statistic	Sig.
P. sativum-R. odorata	-1.333	11.402	-.117	.907
P. sativum-C. papaya	3.000	11.402	.263	.792
P. sativum-M. oleifera	3.167	11.402	.278	.781
P. sativum-T. majus	-8.667	11.402	-.760	.447
P. sativum-C. bursa-pastoris	10.500	11.402	.921	.357
P. sativum-B. orientalis	12.500	11.402	1.096	.273
P. sativum-B. oleracea	19.000	11.402	1.666	.096
P. sativum-I. amara	20.833	11.402	1.827	.068
P. sativum-B. vulgaris	25.000	11.402	2.193	.028
P. sativum-C. pratensis	25.333	11.402	2.222	.026
P. sativum-C. spinosa	30.167	11.402	2.646	.008
P. sativum-A. caucasica	31.833	11.402	2.792	.005
P. sativum-E. cheiri	32.833	11.402	2.880	.004
P. sativum-L. douglasii	34.333	11.402	3.011	.003

P. sativum-A. argenteum	37.500	11.402	3.289	.001
R. odorata-C. papaya	1.667	11.402	.146	.884
R. odorata-M. oleifera	1.833	11.402	.161	.872
R. odorata-T. majus	-7.333	11.402	-.643	.520
R. odorata-C. bursa-pastoris	9.167	11.402	.804	.421
R. odorata-B. orientalis	11.167	11.402	.979	.327
R. odorata-B. oleracea	17.667	11.402	1.549	.121
R. odorata-I. amara	19.500	11.402	1.710	.087
R. odorata-B. vulgaris	23.667	11.402	2.076	.038
R. odorata-C. pratensis	24.000	11.402	2.105	.035
R. odorata-C. spinosa	28.833	11.402	2.529	.011
R. odorata-A. caucasica	30.500	11.402	2.675	.007
R. odorata-E. cheiri	31.500	11.402	2.763	.006
R. odorata-L. douglasii	33.000	11.402	2.894	.004
R. odorata-A. argenteum	36.167	11.402	3.172	.002
C. papaya-M. oleifera	-.167	11.402	-.015	.988
C. papaya-T. majus	-5.667	11.402	-.497	.619
C. papaya-C. bursa-pastoris	7.500	11.402	.658	.511
C. papaya-B. orientalis	9.500	11.402	.833	.405
C. papaya-B. oleracea	16.000	11.402	1.403	.161
C. papaya-I. amara	-17.833	11.402	-1.564	.118
C. papaya-B. vulgaris	22.000	11.402	1.930	.054
C. papaya-C. pratensis	-22.333	11.402	-1.959	.050
C. papaya-C. spinosa	-27.167	11.402	-2.383	.017
C. papaya-A. caucasica	28.833	11.402	2.529	.011
C. papaya-E. cheiri	-29.833	11.402	-2.617	.009
C. papaya-L. douglasii	-31.333	11.402	-2.748	.006
C. papaya-A. argenteum	34.500	11.402	3.026	.002
M. oleifera-T. majus	-5.500	11.402	-.482	.630
M. oleifera-C. bursa-pastoris	7.333	11.402	.643	.520
M. oleifera-B. orientalis	9.333	11.402	.819	.413
M. oleifera-B. oleracea	15.833	11.402	1.389	.165
M. oleifera-I. amara	17.667	11.402	1.549	.121
M. oleifera-B. vulgaris	21.833	11.402	1.915	.056
M. oleifera-C. pratensis	22.167	11.402	1.944	.052
M. oleifera-C. spinosa	27.000	11.402	2.368	.018
M. oleifera-A. caucasica	28.667	11.402	2.514	.012
M. oleifera-E. cheiri	29.667	11.402	2.602	.009

M. oleifera-L. douglasii	31.167	11.402	2.733	.006
M. oleifera-A. argenteum	34.333	11.402	3.011	.003
T. majus-C. bursa-pastoris	1.833	11.402	.161	.872
T. majus-B. orientalis	3.833	11.402	.336	.737
T. majus-B. oleracea	10.333	11.402	.906	.365
T. majus-I. amara	12.167	11.402	1.067	.286
T. majus-B. vulgaris	16.333	11.402	1.433	.152
T. majus-C. pratensis	16.667	11.402	1.462	.144
T. majus-C. spinosa	21.500	11.402	1.886	.059
T. majus-A. caucasica	23.167	11.402	2.032	.042
T. majus-E. cheiri	24.167	11.402	2.120	.034
T. majus-L. douglasii	25.667	11.402	2.251	.024
T. majus-A. argenteum	28.833	11.402	2.529	.011
C. bursa-pastoris-B. orientalis	2.000	11.402	.175	.861
C. bursa-pastoris-B. oleracea	8.500	11.402	.745	.456
C. bursa-pastoris-I. amara	-10.333	11.402	-.906	.365
C. bursa-pastoris-B. vulgaris	14.500	11.402	1.272	.203
C. bursa-pastoris-C. pratensis	-14.833	11.402	-1.301	.193
C. bursa-pastoris-C. spinosa	-19.667	11.402	-1.725	.085
C. bursa-pastoris-A. caucasica	21.333	11.402	1.871	.061
C. bursa-pastoris-E. cheiri	-22.333	11.402	-1.959	.050
C. bursa-pastoris-L. douglasii	-23.833	11.402	-2.090	.037
C. bursa-pastoris-A. argenteum	27.000	11.402	2.368	.018
B. orientalis-B. oleracea	6.500	11.402	.570	.569
B. orientalis-I. amara	-8.333	11.402	-.731	.465
B. orientalis-B. vulgaris	-12.500	11.402	-1.096	.273
B. orientalis-C. pratensis	-12.833	11.402	-1.126	.260
B. orientalis-C. spinosa	-17.667	11.402	-1.549	.121
B. orientalis-A. caucasica	19.333	11.402	1.696	.090
B. orientalis-E. cheiri	-20.333	11.402	-1.783	.075
B. orientalis-L. douglasii	-21.833	11.402	-1.915	.056
B. orientalis-A. argenteum	25.000	11.402	2.193	.028
B. oleracea-I. amara	-1.833	11.402	-.161	.872
B. oleracea-B. vulgaris	-6.000	11.402	-.526	.599
B. oleracea-C. pratensis	-6.333	11.402	-.555	.579
B. oleracea-C. spinosa	-11.167	11.402	-.979	.327
B. oleracea-A. caucasica	12.833	11.402	1.126	.260
B. oleracea-E. cheiri	-13.833	11.402	-1.213	.225

B. oleracea-L. douglasii	-15.333	11.402	-1.345	.179
B. oleracea-A. argenteum	18.500	11.402	1.623	.105
I. amara-B. vulgaris	4.167	11.402	.365	.715
I. amara-C. pratensis	4.500	11.402	.395	.693
I. amara-C. spinosa	9.333	11.402	.819	.413
I. amara-A. caucasica	11.000	11.402	.965	.335
I. amara-E. cheiri	12.000	11.402	1.052	.293
I. amara-L. douglasii	-13.500	11.402	-1.184	.236
I. amara-A. argenteum	16.667	11.402	1.462	.144
B. vulgaris-C. pratensis	-.333	11.402	-.029	.977
B. vulgaris-C. spinosa	-5.167	11.402	-.453	.650
B. vulgaris-A. caucasica	6.833	11.402	.599	.549
B. vulgaris-E. cheiri	-7.833	11.402	-.687	.492
B. vulgaris-L. douglasii	-9.333	11.402	-.819	.413
B. vulgaris-A. argenteum	12.500	11.402	1.096	.273
C. pratensis-C. spinosa	-4.833	11.402	-.424	.672
C. pratensis-A. caucasica	6.500	11.402	.570	.569
C. pratensis-E. cheiri	-7.500	11.402	-.658	.511
C. pratensis-L. douglasii	-9.000	11.402	-.789	.430
C. pratensis-A. argenteum	12.167	11.402	1.067	.286
C. spinosa-A. caucasica	1.667	11.402	.146	.884
C. spinosa-E. cheiri	-2.667	11.402	-.234	.815
C. spinosa-L. douglasii	-4.167	11.402	-.365	.715
C. spinosa-A. argenteum	7.333	11.402	.643	.520
A. caucasica-E. cheiri	-1.000	11.402	-.088	.930
A. caucasica-L. douglasii	-2.500	11.402	-.219	.826
A. caucasica-A. argenteum	5.667	11.402	.497	.619
E. cheiri-L. douglasii	-1.500	11.402	-.132	.895
E. cheiri-A. argenteum	4.667	11.402	.409	.682
L. douglasii-A. argenteum	3.167	11.402	.278	.781

Table S8. Comparison betweeen *P. rapae* and *P. xylostella* for the percentage of eggs laid on the abaxial side of the leaves in the plant species tested (n = 3-96). Data were analyzed using a one-tailed, two-sample test of proportions ($p \leq 0.05$). Significant differences are shown in bold type; n/a: not available, not possible to calculate because oviposition occurred in less than two replicates in at least one of the species.

	Test Statistic, *p*-value
A. argenteum	n/a
A. thaliana	**z=4.61, p≤0.001**
A. caucasica	z=0.06, p=0.476
B. vulgaris	z=0.68, p=0.249
B. oleracea	z=1.26, p=0.103
B. orientalis	z=0.28, p=0.388
C. bursa-pastoris	n/a
C. pratensis	z=0.25, p=0.399
C. papaya	n/a
C. spinosa	z=0.25, p=0.400
E. cheiri	z=0.08, p=0.469
I. amara	z=0.26, p=0.397
L. douglasii	n/a
M. oleifera	n/a
P. sativum	n/a
R. odorata	z=0.60, p=0.274
T. majus	z=1.01, p=0.156

Table S9. Pairwise comparisons in larval survival (LS) between plant species after conducting Kruskal-Wallis test. The test statistic (TS) and *p*-values of the Kruskal-Wallis tests were TS=49.45 and $p \leq 0.001$ and TS=31.64 and $p=0.011$ for *P. rapae* and *P. xylostella*, respectively. The pairwise comparisons among plant species is shown below for *P. rapae* (A) and *P. xylostella* (B). Each row tests the null hypothesis that the Sample 1 and Sample 2 distributions are the same. Asymptotic significances (2-sided tests) are displayed. The significance level is 0.050.

A

Pairwise comparisons of plant species for *P. rapae* LS

Sample 1-Sample 2	Test Statistic	Std. Error	Std. Test Statistic	Sig.
A. argenteum-A. caucasica	.000	9.760	.000	1.000
A. argenteum-B. orientalis	.000	9.760	.000	1.000
A. argenteum-C. bursa-pastoris	.000	9.760	.000	1.000
A. argenteum-C. papaya	.000	9.760	.000	1.000
A. argenteum-C. spinosa	.000	9.760	.000	1.000
A. argenteum-E. cheiri	.000	9.760	.000	1.000
A. argenteum-I. amara	.000	9.760	.000	1.000
A. argenteum-L. douglasii	.000	9.760	.000	1.000
A. argenteum-M. oleifera	.000	9.760	.000	1.000
A. argenteum-P. sativum	.000	9.760	.000	1.000
A. argenteum-T. majus	.000	9.760	.000	1.000
A. argenteum-B. vulgaris	-19.833	9.760	-2.032	.042
A. argenteum-A. thaliana	-23.167	9.760	-2.374	.018
A. argenteum-R. odorata	-27.167	9.760	-2.784	.005
A. argenteum-B. oleracea	-28.667	9.760	-2.937	.003
A. argenteum-C. pratensis	-28.667	9.760	-2.937	.003
A. caucasica-B. orientalis	.000	9.760	.000	1.000
A. caucasica-C. bursa-pastoris	.000	9.760	.000	1.000
A. caucasica-C. papaya	.000	9.760	.000	1.000
A. caucasica-C. spinosa	.000	9.760	.000	1.000
A. caucasica-E. cheiri	.000	9.760	.000	1.000
A. caucasica-I. amara	.000	9.760	.000	1.000
A. caucasica-L. douglasii	.000	9.760	.000	1.000
A. caucasica-M. oleifera	.000	9.760	.000	1.000
A. caucasica-P. sativum	.000	9.760	.000	1.000

A. caucasica-T. majus	.000	9.760	.000	1.000
A. caucasica-B. vulgaris	-19.833	9.760	-2.032	.042
A. caucasica-A. thaliana	-23.167	9.760	-2.374	.018
A. caucasica-R. odorata	-27.167	9.760	-2.784	.005
A. caucasica-B. oleracea	-28.667	9.760	-2.937	.003
A. caucasica-C. pratensis	-28.667	9.760	-2.937	.003
L. douglasii-T. majus	.000	9.760	.000	1.000
B. orientalis-A. thaliana	23.167	9.760	2.374	.018
M. oleifera-P. sativum	.000	9.760	.000	1.000
C. bursa-pastoris-A. thaliana	23.167	9.760	2.374	.018
C. papaya-A. thaliana	23.167	9.760	2.374	.018
M. oleifera-R. odorata	-27.167	9.760	-2.784	.005
C. spinosa-A. thaliana	23.167	9.760	2.374	.018
E. cheiri-A. thaliana	23.167	9.760	2.374	.018
I. amara-A. thaliana	23.167	9.760	2.374	.018
L. douglasii-A. thaliana	23.167	9.760	2.374	.018
M. oleifera-A. thaliana	23.167	9.760	2.374	.018
P. sativum-A. thaliana	23.167	9.760	2.374	.018
M. oleifera-T. majus	.000	9.760	.000	1.000
T. majus-A. thaliana	23.167	9.760	2.374	.018
B. orientalis-B. oleracea	28.667	9.760	2.937	.003
P. sativum-R. odorata	-27.167	9.760	-2.784	.005
C. bursa-pastoris-B. oleracea	28.667	9.760	2.937	.003
C. papaya-B. oleracea	28.667	9.760	2.937	.003
P. sativum-T. majus	.000	9.760	.000	1.000
C. spinosa-B. oleracea	28.667	9.760	2.937	.003
E. cheiri-B. oleracea	28.667	9.760	2.937	.003
I. amara-B. oleracea	28.667	9.760	2.937	.003
L. douglasii-B. oleracea	28.667	9.760	2.937	.003
M. oleifera-B. oleracea	28.667	9.760	2.937	.003
P. sativum-B. oleracea	28.667	9.760	2.937	.003
T. majus-R. odorata	27.167	9.760	2.784	.005
T. majus-B. oleracea	28.667	9.760	2.937	.003
B. orientalis-C. bursa-pastoris	.000	9.760	.000	1.000
B. orientalis-C. papaya	.000	9.760	.000	1.000
B. orientalis-C. spinosa	.000	9.760	.000	1.000
B. orientalis-E. cheiri	.000	9.760	.000	1.000
B. orientalis-I. amara	.000	9.760	.000	1.000

B. orientalis-L. douglasii	.000	9.760	.000	1.000
B. orientalis-M. oleifera	.000	9.760	.000	1.000
B. orientalis-P. sativum	.000	9.760	.000	1.000
B. orientalis-T. majus	.000	9.760	.000	1.000
B. orientalis-B. vulgaris	-19.833	9.760	-2.032	.042
B. orientalis-R. odorata	-27.167	9.760	-2.784	.005
B. orientalis-C. pratensis	-28.667	9.760	-2.937	.003
C. bursa-pastoris-B. vulgaris	19.833	9.760	2.032	.042
C. papaya-B. vulgaris	19.833	9.760	2.032	.042
C. spinosa-B. vulgaris	19.833	9.760	2.032	.042
E. cheiri-B. vulgaris	19.833	9.760	2.032	.042
I. amara-B. vulgaris	19.833	9.760	2.032	.042
L. douglasii-B. vulgaris	19.833	9.760	2.032	.042
M. oleifera-B. vulgaris	19.833	9.760	2.032	.042
P. sativum-B. vulgaris	19.833	9.760	2.032	.042
T. majus-B. vulgaris	19.833	9.760	2.032	.042
C. bursa-pastoris-C. papaya	.000	9.760	.000	1.000
C. bursa-pastoris-C. spinosa	.000	9.760	.000	1.000
C. bursa-pastoris-E. cheiri	.000	9.760	.000	1.000
C. bursa-pastoris-I. amara	.000	9.760	.000	1.000
C. bursa-pastoris-L. douglasii	.000	9.760	.000	1.000
C. bursa-pastoris-M. oleifera	.000	9.760	.000	1.000
C. bursa-pastoris-P. sativum	.000	9.760	.000	1.000
C. bursa-pastoris-T. majus	.000	9.760	.000	1.000
C. bursa-pastoris-R. odorata	-27.167	9.760	-2.784	.005
C. bursa-pastoris-C. pratensis	-28.667	9.760	-2.937	.003
C. papaya-C. spinosa	.000	9.760	.000	1.000
C. papaya-E. cheiri	.000	9.760	.000	1.000
C. papaya-I. amara	.000	9.760	.000	1.000
C. papaya-L. douglasii	.000	9.760	.000	1.000
C. papaya-M. oleifera	.000	9.760	.000	1.000
C. papaya-P. sativum	.000	9.760	.000	1.000
C. papaya-T. majus	.000	9.760	.000	1.000
C. papaya-R. odorata	-27.167	9.760	-2.784	.005
C. papaya-C. pratensis	-28.667	9.760	-2.937	.003
C. spinosa-C. pratensis	28.667	9.760	2.937	.003
E. cheiri-C. pratensis	28.667	9.760	2.937	.003
I. amara-C. pratensis	28.667	9.760	2.937	.003

L. douglasii-C. pratensis	28.667	9.760	2.937	.003
M. oleifera-C. pratensis	28.667	9.760	2.937	.003
P. sativum-C. pratensis	28.667	9.760	2.937	.003
T. majus-C. pratensis	28.667	9.760	2.937	.003
C. spinosa-E. cheiri	.000	9.760	.000	1.000
C. spinosa-I. amara	.000	9.760	.000	1.000
C. spinosa-L. douglasii	.000	9.760	.000	1.000
C. spinosa-M. oleifera	.000	9.760	.000	1.000
C. spinosa-P. sativum	.000	9.760	.000	1.000
C. spinosa-T. majus	.000	9.760	.000	1.000
C. spinosa-R. odorata	-27.167	9.760	-2.784	.005
E. cheiri-I. amara	.000	9.760	.000	1.000
E. cheiri-L. douglasii	.000	9.760	.000	1.000
E. cheiri-M. oleifera	.000	9.760	.000	1.000
E. cheiri-P. sativum	.000	9.760	.000	1.000
E. cheiri-T. majus	.000	9.760	.000	1.000
E. cheiri-R. odorata	-27.167	9.760	-2.784	.005
I. amara-L. douglasii	.000	9.760	.000	1.000
I. amara-M. oleifera	.000	9.760	.000	1.000
I. amara-P. sativum	.000	9.760	.000	1.000
I. amara-T. majus	.000	9.760	.000	1.000
I. amara-R. odorata	-27.167	9.760	-2.784	.005
L. douglasii-M. oleifera	.000	9.760	.000	1.000
L. douglasii-P. sativum	.000	9.760	.000	1.000
L. douglasii-R. odorata	-27.167	9.760	-2.784	.005
B. vulgaris-A. thaliana	3.333	9.760	.342	.733
B. vulgaris-R. odorata	-7.333	9.760	-.751	.452
B. vulgaris-B. oleracea	8.833	9.760	.905	.365
B. vulgaris-C. pratensis	-8.833	9.760	-.905	.365
A. thaliana-R. odorata	-4.000	9.760	-.410	.682
A. thaliana-B. oleracea	-5.500	9.760	-.564	.573
A. thaliana-C. pratensis	-5.500	9.760	-.564	.573
R. odorata-B. oleracea	1.500	9.760	.154	.878
R. odorata-C. pratensis	1.500	9.760	.154	.878
B. oleracea-C. pratensis	.000	9.760	.000	1.000

B

Pairwise comparisons of plant species for *P. xylostella* LS

Sample 1-Sample 2	Test Statistic	Std. Error	Std. Test Statistic	Sig.
C. papaya-C. spinosa	-6.333	13.407	-.472	.637
C. papaya-M. oleifera	-7.500	12.541	-.598	.550
C. papaya-B. orientalis	12.667	13.407	.945	.345
C. papaya-E. cheiri	-32.125	12.541	-2.562	.010
B. vulgaris-A. argenteum	17.000	12.541	1.356	.175
C. papaya-A. argenteum	17.000	12.541	1.356	.175
C. papaya-C. pratensis	-38.667	13.407	-2.884	.004
C. papaya-L. douglasii	-38.667	13.407	-2.884	.004
P. sativum-C. bursa-pastoris	16.333	13.407	1.218	.223
P. sativum-C. pratensis	38.667	13.407	2.884	.004
B. vulgaris-I. amara	-27.750	12.541	-2.213	.027
P. sativum-C. spinosa	6.333	13.407	.472	.637
B. vulgaris-E. cheiri	-32.125	12.541	-2.562	.010
P. sativum-A. argenteum	17.000	12.541	1.356	.175
C. papaya-C. bursa-pastoris	16.333	13.407	1.218	.223
C. papaya-I. amara	-27.750	12.541	-2.213	.027
B. vulgaris-B. orientalis	12.667	13.407	.945	.345
P. sativum-B. oleracea	26.333	13.407	1.964	.050
B. vulgaris-A. caucasica	15.500	12.541	1.236	.216
B. vulgaris-C. bursa-pastoris	-16.333	13.407	-1.218	.223
P. sativum-I. amara	27.750	12.541	2.213	.027
C. papaya-A. caucasica	15.500	12.541	1.236	.216
B. vulgaris-C. papaya	.000	13.407	.000	1.000
P. sativum-M. oleifera	7.500	12.541	.598	.550
P. sativum-L. douglasii	38.667	13.407	2.884	.004
B. vulgaris-C. pratensis	-38.667	13.407	-2.884	.004
P. sativum-A. thaliana	30.667	13.407	2.287	.022
B. vulgaris-B. oleracea	26.333	13.407	1.964	.050
P. sativum-A. caucasica	15.500	12.541	1.236	.216
C. papaya-B. oleracea	26.333	13.407	1.964	.050
P. sativum-B. orientalis	12.667	13.407	.945	.345
B. vulgaris-C. spinosa	-6.333	13.407	-.472	.637

B. vulgaris-R. odorata	-10.750	12.541	-.857	.391
B. vulgaris-T. majus	-16.200	11.992	-1.351	.177
B. vulgaris-A. thaliana	30.667	13.407	2.287	.022
C. papaya-A. thaliana	30.667	13.407	2.287	.022
B. vulgaris-P. sativum	.000	13.407	.000	1.000
B. vulgaris-M. oleifera	-7.500	12.541	-.598	.550
B. vulgaris-L. douglasii	-38.667	13.407	-2.884	.004
C. papaya-P. sativum	.000	13.407	.000	1.000
C. papaya-R. odorata	-10.750	12.541	-.857	.391
C. papaya-T. majus	-16.200	11.992	-1.351	.177
P. sativum-R. odorata	-10.750	12.541	-.857	.391
P. sativum-T. majus	-16.200	11.992	-1.351	.177
P. sativum-E. cheiri	32.125	12.541	2.562	.010
C. spinosa-M. oleifera	-1.167	12.541	-.093	.926
C. spinosa-R. odorata	-4.417	12.541	-.352	.725
C. spinosa-B. orientalis	6.333	13.407	.472	.637
C. spinosa-A. caucasica	9.167	12.541	.731	.465
C. spinosa-T. majus	-9.867	11.992	-.823	.411
C. spinosa-C. bursa-pastoris	10.000	13.407	.746	.456
C. spinosa-A. argenteum	10.667	12.541	.851	.395
C. spinosa-B. oleracea	20.000	13.407	1.492	.136
C. spinosa-I. amara	-21.417	12.541	-1.708	.088
C. spinosa-A. thaliana	24.333	13.407	1.815	.070
C. spinosa-E. cheiri	-25.792	12.541	-2.057	.040
C. spinosa-C. pratensis	32.333	13.407	2.412	.016
C. spinosa-L. douglasii	-32.333	13.407	-2.412	.016
M. oleifera-R. odorata	-3.250	11.611	-.280	.780
M. oleifera-B. orientalis	5.167	12.541	.412	.680
M. oleifera-A. caucasica	8.000	11.611	.689	.491
M. oleifera-T. majus	-8.700	11.015	-.790	.430
M. oleifera-C. bursa-pastoris	8.833	12.541	.704	.481
M. oleifera-A. argenteum	9.500	11.611	.818	.413
M. oleifera-B. oleracea	18.833	12.541	1.502	.133
M. oleifera-I. amara	20.250	11.611	1.744	.081
M. oleifera-A. thaliana	23.167	12.541	1.847	.065
M. oleifera-E. cheiri	24.625	11.611	2.121	.034
M. oleifera-C. pratensis	31.167	12.541	2.485	.013
M. oleifera-L. douglasii	31.167	12.541	2.485	.013

R. odorata-B. orientalis	1.917	12.541	.153	.879
R. odorata-A. caucasica	4.750	11.611	.409	.682
R. odorata-T. majus	-5.450	11.015	-.495	.621
R. odorata-C. bursa-pastoris	5.583	12.541	.445	.656
R. odorata-A. argenteum	6.250	11.611	.538	.590
R. odorata-B. oleracea	15.583	12.541	1.243	.214
R. odorata-I. amara	17.000	11.611	1.464	.143
R. odorata-A. thaliana	19.917	12.541	1.588	.112
R. odorata-E. cheiri	21.375	11.611	1.841	.066
R. odorata-C. pratensis	27.917	12.541	2.226	.026
R. odorata-L. douglasii	27.917	12.541	2.226	.026
B. orientalis-A. caucasica	2.833	12.541	.226	.821
B. orientalis-T. majus	-3.533	11.992	-.295	.768
B. orientalis-C. bursa-pastoris	-3.667	13.407	-.273	.784
B. orientalis-A. argenteum	4.333	12.541	.346	.730
B. orientalis-B. oleracea	13.667	13.407	1.019	.308
B. orientalis-I. amara	-15.083	12.541	-1.203	.229
B. orientalis-A. thaliana	18.000	13.407	1.343	.179
B. orientalis-E. cheiri	-19.458	12.541	-1.552	.121
B. orientalis-C. pratensis	-26.000	13.407	-1.939	.052
B. orientalis-L. douglasii	-26.000	13.407	-1.939	.052
A. caucasica-T. majus	-.700	11.015	-.064	.949
A. caucasica-C. bursa-pastoris	-.833	12.541	-.066	.947
A. caucasica-A. argenteum	1.500	11.611	.129	.897
A. caucasica-B. oleracea	-10.833	12.541	-.864	.388
A. caucasica-I. amara	-12.250	11.611	-1.055	.291
A. caucasica-A. thaliana	-15.167	12.541	-1.209	.227
A. caucasica-E. cheiri	-16.625	11.611	-1.432	.152
A. caucasica-L. douglasii	-23.167	12.541	-1.847	.065
A. caucasica-C. pratensis	-23.167	12.541	-1.847	.065
T. majus-C. bursa-pastoris	.133	11.992	.011	.991
T. majus-A. argenteum	.800	11.015	.073	.942
T. majus-B. oleracea	10.133	11.992	.845	.398
T. majus-I. amara	11.550	11.015	1.049	.294
T. majus-A. thaliana	14.467	11.992	1.206	.228
T. majus-E. cheiri	15.925	11.015	1.446	.148
T. majus-C. pratensis	22.467	11.992	1.874	.061
T. majus-L. douglasii	22.467	11.992	1.874	.061

C. bursa-pastoris-A. argenteum	.667	12.541	.053	.958
C. bursa-pastoris-B. oleracea	10.000	13.407	.746	.456
C. bursa-pastoris-I. amara	-11.417	12.541	-.910	.363
C. bursa-pastoris-A. thaliana	14.333	13.407	1.069	.285
C. bursa-pastoris-E. cheiri	-15.792	12.541	-1.259	.208
C. bursa-pastoris-C. pratensis	-22.333	13.407	-1.666	.096
C. bursa-pastoris-L. douglasii	-22.333	13.407	-1.666	.096
A. argenteum-B. oleracea	-9.333	12.541	-.744	.457
A. argenteum-I. amara	-10.750	11.611	-.926	.355
A. argenteum-A. thaliana	-13.667	12.541	-1.090	.276
A. argenteum-E. cheiri	-15.125	11.611	-1.303	.193
A. argenteum-L. douglasii	-21.667	12.541	-1.728	.084
A. argenteum-C. pratensis	-21.667	12.541	-1.728	.084
B. oleracea-I. amara	-1.417	12.541	-.113	.910
B. oleracea-A. thaliana	4.333	13.407	.323	.747
B. oleracea-E. cheiri	-5.792	12.541	-.462	.644
B. oleracea-L. douglasii	-12.333	13.407	-.920	.358
B. oleracea-C. pratensis	-12.333	13.407	-.920	.358
I. amara-A. thaliana	2.917	12.541	.233	.816
I. amara-E. cheiri	4.375	11.611	.377	.706
I. amara-C. pratensis	10.917	12.541	.870	.384
I. amara-L. douglasii	-10.917	12.541	-.870	.384
A. thaliana-E. cheiri	-1.458	12.541	-.116	.907
A. thaliana-L. douglasii	-8.000	13.407	-.597	.551
A. thaliana-C. pratensis	-8.000	13.407	-.597	.551
E. cheiri-L. douglasii	-6.542	12.541	-.522	.602
E. cheiri-C. pratensis	6.542	12.541	.522	.602
C. pratensis-L. douglasii	.000	13.407	.000	1.000

Table S10. Origin of the seeds of the plant species tested.

Species	Provider of seeds, city, country
Alyssum argenteum All.	Jelitto, Schwarmstedt, Germany
Arabis caucasica Willd.	B & T World Seeds, Aigues-Vives, France
Arabidopsis thaliana (L.) Heynh.	European Arabidopsis Stock Center, Nottingham University, Loughborough, UK
Barbarea vulgaris R.Br.	Dr. Niels Agerbirk, University of Copenhagen, Copenhagen, Denmark
Brassica oleracea var. *capitata* L.	B & T World Seeds, Aigues-Vives, France
Bunias orientalis L.	B & T World Seeds, Aigues-Vives, France
Capsella bursa-pastoris (L.) Medik.	B & T World Seeds, Aigues-Vives, France
Cardamine pratensis L.	Rühlemann´s, Horstedt, Germany
Carica papaya L.	B & T World Seeds, Aigues-Vives, France
Cleome spinosa L.	B & T World Seeds, Aigues-Vives, France
Erysimum cheiri (L.) Crantz	B & T World Seeds, Aigues-Vives, France
Iberis amara L.	B & T World Seeds, Aigues-Vives, France
Limnanthes douglasii R. Br.	B & T World Seeds, Aigues-Vives, France
Moringa oleifera Lam.	B & T World Seeds, Aigues-Vives, France
Pisum sativum L.	B & T World Seeds, Aigues-Vives, France
Reseda odorata L.	B & T World Seeds, Aigues-Vives, France
Tropaeolum majus L.	B & T World Seeds, Aigues-Vives, France

Figure S1. CATPCA plots showing the relationship between oviposition preference index (OPI) (A), total oviposition (TO) (B), and larval survival (LS) (C) and total glucosinolate content (TOT), content of aliphatic glucosinolates with sulfur-containing side chains (AS), other aliphatic glucosinolates (AO), benzenic glucosinolates (BEN), and indolic glucosinolates (IN) in the plant species tested. The relationship of OPI, TO, and LS with glucosinolate richness (*S*), Shannon´s diversity index for the four glucosinolate classes (H_A), Shannon´s diversity index for the relative concentrations of all individual glucosinolates (H_B), and chemical complexity index for glucosinolates (GCI) is also shown.

A

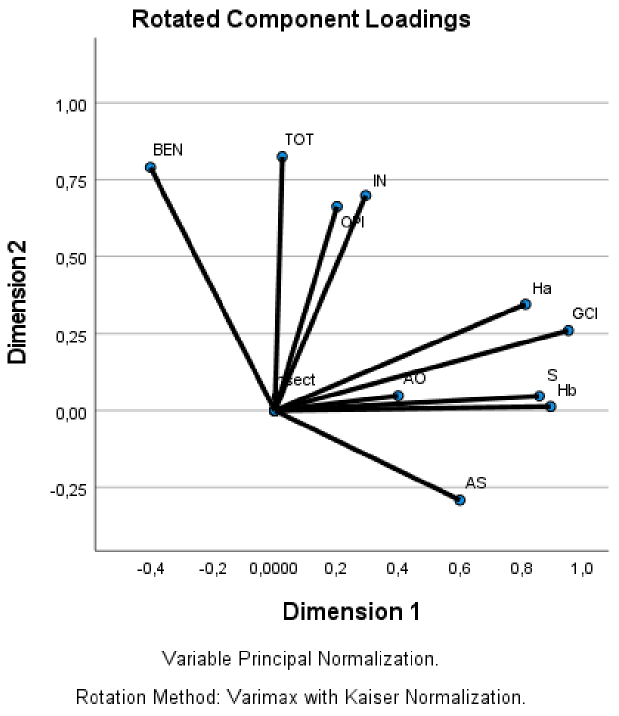

B

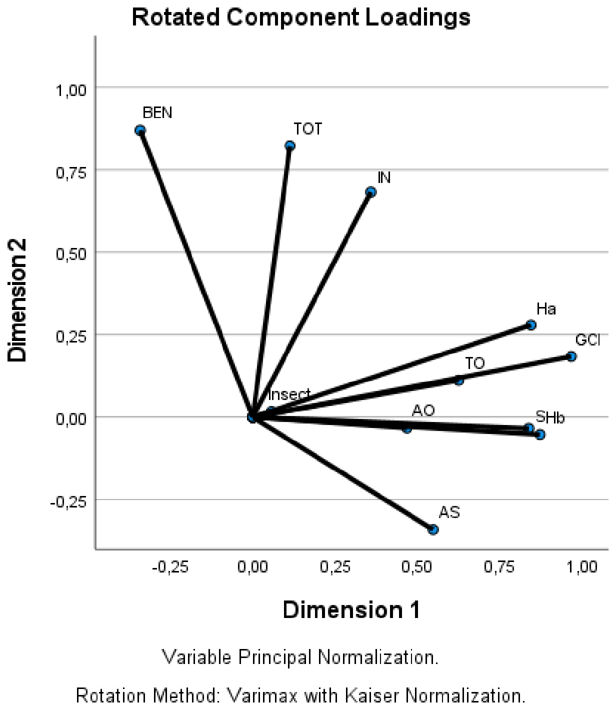

Rotated Component Loadings
Dimension 2
Dimension 1
1,00
0,75
0,50
0,25
0,00
-0,25
-0,25
0,00
0,25
0,50
0,75
1,00
BEN
TOT
IN
Ha
GCI
TO
Insect
AO
SHb
AS
Variable Principal Normalization.
Rotation Method: Varimax with Kaiser Normalization.

C

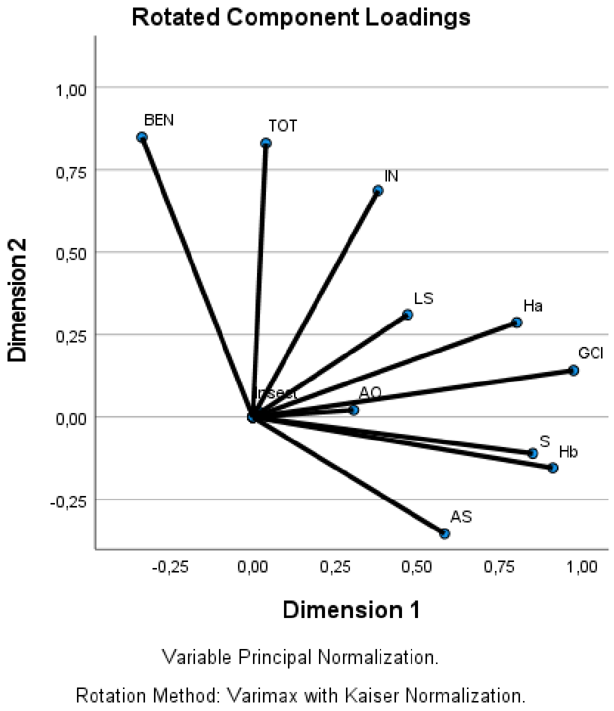

Reference Cited

37. Badenes-Pérez. F.R.; Gershenzon. J.; Heckel. D.G. Plant glucosinolate content increases susceptibility to diamondback moth (Lepidoptera: Plutellidae) regardless of its diet. *Journal of Pest Science* **2020**. *93*. 491–506.
